# Polymorph sampling with coupling to extended variables: enhanced sampling of polymorph energy landscapes and free energy perturbation of polymorph ensembles

**DOI:** 10.1107/S205252062400132X

**Published:** 2024-10-15

**Authors:** Eric J. Chan, Mark E. Tuckerman

**Affiliations:** a Chemistry Department Curtin University Bentley WA 6102 Australia; b Department of Chemistry New York University New York City NY 10003 USA; c Courant Institute of Mathematical Science, New York University, New York City, NY, 10003, USA; d New York University-East China Normal University Center for Computational Chemistry at NYU Shanghai 3663 Zhongshan Road North Shanghai200062 China; CSIR–National Chemical Laboratory, India

**Keywords:** crystal structure prediction (CSP), polymorphism, enhanced sampling, lattice ensemble free energies

## Abstract

A novel approach to computationally enhance the sampling of molecular crystal structures is proposed and tested. This method is based on the use of extended variables coupled to a Monte Carlo based crystal polymorph generator. Inspired by the established technique of quasi-random sampling of polymorphs using the rigid molecule constraint, this approach represents molecular clusters as extended variables within a thermal reservoir. Polymorph unit-cell variables are generated using pseudo-random sampling. Within this framework, a harmonic coupling between the extended variables and polymorph configurations is established. The extended variables remain fixed during the inner loop dedicated to polymorph sampling, enforcing a stepwise propagation of the extended variables to maintain system exploration. The final processing step results in a polymorph energy landscape, where the raw structures sampled to create the extended variable trajectory are re-optimized without the thermal coupling term. The foundational principles of this approach are described and its effectiveness using both a Metropolis Monte Carlo type algorithm and modifications that incorporate replica exchange is demonstrated. A comparison is provided with pseudo-random sampling of polymorphs for the molecule coumarin. The choice to test a design of this algorithm as relevant for enhanced sampling of crystal structures was due to the obvious relation between molecular structure variables and corresponding crystal polymorphs as representative of the inherent vapor to crystal transitions that exist in nature. Additionally, it is shown that the trajectories of extended variables can be harnessed to extract fluctuation properties that can lead to valuable insights. A novel thermodynamic variable is introduced: the free energy difference between ensembles of *Z*′ = 1 and *Z*′ = 2 crystal polymorphs.

## Abstract 2

A design is tested for a polymorph search algorithm relevant for enhanced sampling of crystal structures based on the relation between internal molecular structure variables and corresponding crystal polymorphs as representative of the inherent vapor to crystal transitions that exist in nature. Molecules are represented as extended variables within a thermal reservoir. Unit-cell variables are generated using pseudo-random sampling incorporating a harmonic coupling to extended variables.

## 1. Introduction

*In-silico* crystal structure prediction (CSP) has gained significant interest among material engineers, chemical control specialists, and solid-state organic chemists (Chan *et al.*, 2021 ▸; Davey & Garside, 2000 ▸; Desiraju, 1989 ▸, 2001 ▸; Hartman, 1973 ▸; Mullin, 2001 ▸; Price, 2013 ▸, 2004 ▸). However, the precise design of molecular building blocks for targeted packing motifs and desired physical properties remains a challenge in crystal engineering (Bernstein, 2008 ▸, 2002 ▸; Dunitz, 1995 ▸; Gavezzotti, 2006 ▸; Kitaigorodskiy *et al.*, 1965 ▸; Kitaigorosky, 1973 ▸). This is due not only to the complex physical laws governing the packing of molecular crystals but also to the role of crystallization kinetics and factors such as nucleation and growth, necessitating practical experimentation as the primary means of design. While new approaches to CSP continue to emerge (Bier *et al.*, 2021 ▸; Day *et al.*, 2003 ▸; Neumann *et al.*, 2008 ▸; Price, 2018 ▸; Reilly *et al.*, 2016 ▸; Schneider *et al.*, 2016 ▸; Yu & Tuckerman, 2011 ▸; Zhu *et al.*, 2014 ▸), the most successful methods often remain closely guarded industrial secrets (Neumann, 2008 ▸; Hunnisett, Nyman *et al.*, 2024 ▸; Hunnisett, Francia *et al.*, 2024 ▸).

Despite the wealth of crystal data in the Cambridge Structural Database (CSD), many reliable CSP approaches still rely on energy-based techniques and configurational sampling, rather than being data-driven. While machine learning has made significant strides in predicting protein structures (Jumper *et al.*, 2021 ▸; Silver *et al.*, 2018 ▸, 2016 ▸), the application of similar breakthroughs in CSP remains a challenge. CSP primarily relies on computational chemistry and molecular simulation techniques (Allen & Tildesley, 1987 ▸; Frenkel & Smit, 2002 ▸; Hermann *et al.*, 2017 ▸; Parr & Weitao, 1995 ▸; Tuckerman, 2010 ▸) to bridge the gap between theory and experimental evidence.

Simulation-driven CSP methods focus on two main objectives: polymorph sampling and ranking stability. Polymorph sampling involves configuration sampling algorithms that require a molecular structure as input, which is the primary topic of this report. Ranking stability aims to accurately predict energy differences between pre-generated polymorph configurations and benefits from insights into crystal nucleation and growth (Case *et al.*, 2016 ▸; Reilly *et al.*, 2016 ▸; Yang & Day, 2021*a* ▸,*b* ▸; Hermann *et al.*, 2017 ▸; Hoja *et al.*, 2017 ▸; Wengert *et al.*, 2021 ▸; Rossi *et al.*, 2016 ▸).

Polymorph sampling methods frequently utilize Monte Carlo (MC) sampling, basin hopping (BH), molecular dynamics (MD) or evolutionary algorithms (Neumann *et al.*, 2008 ▸; Price, 2004 ▸; Yu & Tuckerman, 2011 ▸; Rosso *et al.*, 2002 ▸; Sobol, 1977 ▸; Zhu *et al.*, 2014 ▸; Bier *et al.*, 2021 ▸). Most MC methods such as BH or Sobol sampling involve a subsequent molecular energy optimization from an higher energy test configuration to identify local minima. A key question pertains to how the probability of generating a structure correlates with a compound’s intrinsic ability to crystallize in nature.

Effective and efficient polymorph sampling algorithms must adapt as molecular systems grow in complexity, which may involve an increased number of torsional degrees of freedom or more molecules in the asymmetric unit (*Z*′). *In silico* polymorph screening can often be incomplete (Case *et al.*, 2016 ▸; Sobol, 1977 ▸). CSP faces the challenge of dimensionality as the number of configuration variables increases, making exhaustive searches less feasible. To address this challenge, low-*Z*′ values are often used, and CSP employs pseudo-random (PR) or quasi-random (QR) sampling methods (van Eijck & Kroon, 1999 ▸; Case *et al.*, 2016 ▸), or enhanced sampling schemes (Hasenbusch & Schaefer, 2010 ▸; Laio & Parrinello, 2002 ▸; Liu *et al.*, 2005 ▸; Yu & Tuckerman, 2011 ▸). These techniques expedite the exploration of the configuration space, ensuring adequate sampling of a wide range of crystal structures and polymorphs.

Polymorphs often exhibit complex and diverse structures, and efficiently sampling them within a practical time frame in computational simulations can be challenging. The systems themselves are not inherently non-ergodic, but achieving ergodicity within a reasonable time frame presents a formidable challenge. This challenge also is primarily addressed with enhanced sampling techniques.

This report introduces an enhanced sampling approach, adapted from QR, BH and temperature-accelerated methods, with similarities to umbrella sampling. The method involves stepwise propagation of molecular coordinates represented by extended variables (EVs) in a heat bath, allowing a broader distribution of possible unit cells to be randomly sampled at each step. EVs are reference variables acting as a tool and, as described later, are not necessarily atomic coordinates. During each step, the EVs are held fixed, which biases a pseudo-random polymorph sampling stage. This method is referred to as ‘Extended Variable Coupled to Crystal Polymorph Monte Carlo’ sampling (EVCCPMC or EVCCP).

The reasoning to design and test the EVCCP algorithm for the possibility of enhanced sampling of crystal structures was due to the obvious relation between extended variables being representative of a molecular configuration in the gaseous phase and the statistically biased generation of corresponding polymorphs being representative of inherent vapor to crystal transitions that can exist in nature.

This report aims to introduce and demonstrate EVCCP sampling conceptually within the context of CSP. EVCCP was trialed as part of a recent blind test (Hunnisett, Nyman *et al.*, 2024 ▸). The specific focus of this report is to demonstrate a modification that enables replica exchange, known as the EVCCP modified replica exchange (EVCCPMRE) approach. It is worth noting that this modification conceptually allows for the exchange of non-ordinal extended variables, such as those associated with space group symmetry or *Z*′. However, this is outside the scope of this report and the authors intend to conduct a comprehensive study of EVCCPMRE, particularly focusing on the exchange of space group as a variable, separately as part of a future investigation.

Within the EVCCP framework, EVs exhibit ergodic behavior. This enables the calculation of thermodynamic properties pertaining to ensembles of crystal polymorph configurations. Specifically, it facilitates the calculation of Free Energy Differences (FED) between variables, such as stoichiometric ratios or different values of *Z*′. The latter serves as a qualitative measure of a molecule’s propensity to form a polymorph with a specific *Z*′, distinct from a comparison of selected minimum energy polymorphs.

The system of coumarin polymorphs (Shtukenberg *et al.*, 2017 ▸) was used as the benchmark compound for this investigation and comparisons are made between EVCCP and vanilla pseudo-random polymorph generation (van Eijck & Kroon, 1999 ▸). Coumarin was deemed ideal because it is well suited for rigid body approximation and there are experimentally known forms with different *Z*′ that crystallize in the same space group.

## 2. Method description

The EVCCP framework considers two independent atomic systems that describe identical sets of components (*i.e.* the cluster of *Z*′ molecules). One system represents the crystal polymorph and the other is a reference system containing extended variables (EVs) (Abrams & Tuckerman, 2008 ▸; Laio & Parrinello, 2002 ▸; Maragliano & Vanden-Eijnden, 2006 ▸; Ciccotti & Meloni, 2011 ▸). To elaborate, if the desire is to sample crystal polymorphs with *Z*′ = 4 then the EVs would represent an isolated cluster of four molecules in the gas phase. Both systems contain atomic coordinates (**R**_*i*_), where **R** ∈ 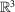 and the subscript *i* represents the *i*th atom in either system.

In EVCCP, the atomic positional coordinates (**R**_*i*_) are mapped onto collective variables (CVs) for molecular centers and orientations (Euler angles). A matrix is used to describe the unit-cell parameters for the crystal polymorph (*i.e.* parallelepiped).

**Figure 1:**
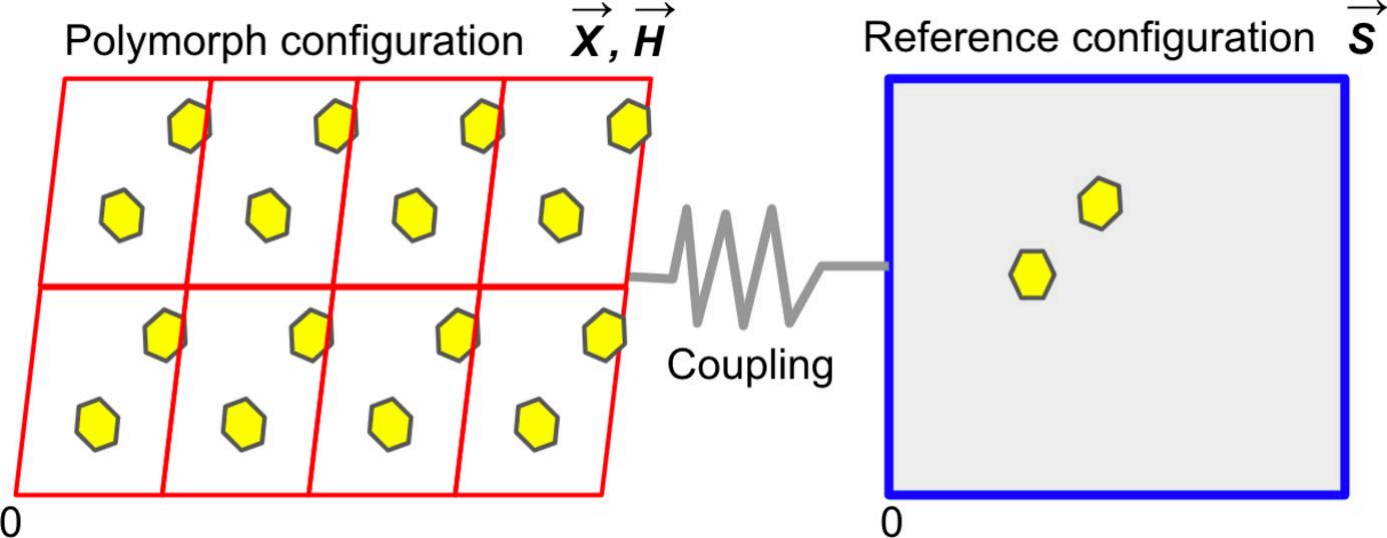
Schematic representation of the crystal polymorph and reference systems in the extended variable coupled to crystal polymorph Monte Carlo (EVCCPMC) scheme. In both systems, the components are identical sets of (extended) variables – **X** or **S** – illustrated using yellow six-membered rings. Red parallelograms are the unit-cell parameters of a crystal (**H**), in contrast with the reference system (blue orthogonal box). The gray spring connecting the two systems represents a harmonic coupling [see equation (2)]. As shown in this diagram, the state of the (extended) variables between systems need not be the same, but will be similar as a result of coupling.

An orthogonal simulation box is used for the reference system that has a volume much greater than the volume occupied by all the atoms. **X** ∈ 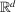 is a vector of coordinates with dimensionality (*d*) representing the CVs in the crystal system (*d* = *Z*′ × 6) and **H** is a vector representing the unit-cell parameters. **H** ∈ 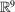 are the vector coordinates for a parallelepiped or unit cell. **H** ≡ {**a**, **b**, **c**}, where **a**, **b** and **c** are the unit-cell vectors in Å. **S** are EVs which correspond with **X** (see Fig. 1 ▸).

The partition function [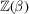] describing the coupled systems is
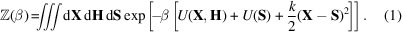
In equation (1), *U*(**X**, **H**) is the potential energy surface (PES) of the crystal polymorphs, *U*(**S**) is the reference system potential and 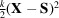 is a harmonic coupling term with a spring constant (*k*). For simplicity, in this study the reference system is chosen to be not self-interacting [*i.e.U*(**S**) = 0]. However, this is not at all a strict requirement and might be exploited for further investigation. The combined potential of the two systems is initially represented using 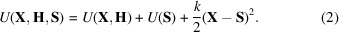
In EVCCP, the evolution of the polymorph system is adiabatically (quasi-static) decoupled from that of the reference system because each **X** and **H** are evaluated while **S** remains unchanged and vice-versa, *i.e.* the evolution or change of either system is subject to largely separate characteristic time-scales. If *k* = 0, the **X** and **H** are obtained by a minimization of the polymorph structure energy *U*(**X**, **H**) from some initial configuration (**X**_0_, **H**_0_). This final configuration will also have a generation probability *P*(**X**, **H**). Instead, *k* > 0 and **X**_0_ are instantiated with some reference coordinate **S** and independently 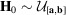, where **a** and **b** are limits on unit-cell vectors defined to specify a range of polymorph densities. The configurational energy currently undergoing minimization is denoted as *U*(**X**, **H**|**S**). This notation is employed here to represent the potential associated with the joint probability [*i.e.P*(**S**)*P(**X**, **H**|**S**) = *P*(**X**, **H**, **S**)], with *U*(**X**, **H**|**S**) specifically emphasizing the fixed nature of **S**. Exploiting the conditional probability *P*(**X**, **H**|**S**) is a conceptual underpinning of EVCCP. In the next section estimates for *P*(**X**, **H**|**S**) are made by statistical inference.

**Figure 2:**
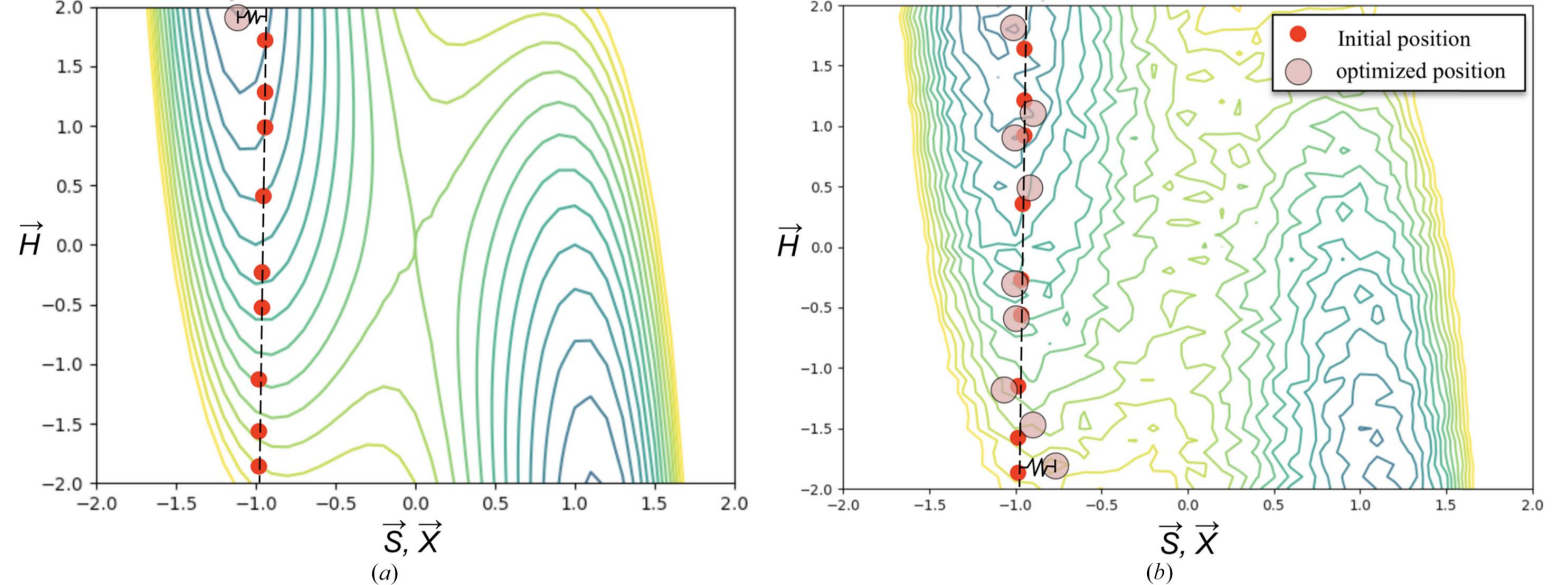
Schematic representation of the mini-batch polymorph generation workflow in EVCCP. (*a*) Colored contours represent a smooth potential energy surface (PES) function *U*(**X**, **H**) and (*b*) is the same surface only roughed with arbitrary noise to create pockets of local minima. In both plots, red circles are the *M* initial points which have the same reference EV **S** indicated by the vertical black dashed line. For the initial positions, 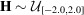 with **X** = **S**. Pink circles indicate the final coordinates. The difference between **S** and **X** (highlighted for one point with a black spring) will make a contribution to *U*(**X**, **H**|**S**) via a harmonic coupling term [see equation (3)]. When the optimization is performed on the smooth surface all points end at the same minimum. In contrast, on the roughed surface the mini-batch global minimum *U*_min_ will be the best approximation for the optimal solution.

**S** are stepwise propagated with temperature (*T*)^1^ according to the metropolis MC updating scheme (Metropolis *et al.*, 1953 ▸). The configuration energy used for these updates (detailed in Appendix *A*[App appa]) is given by 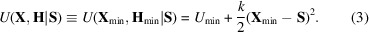
Here *U*_min_ and **X**_min_ are the respective energy and CV coordinate of the polymorph that was generated from a subset number of polymorphs (*M*) generated each EVCCP step which share the same fixed **S** coordinate (essentially argmin[*U*(**X**, **H**, **S**)]). To determine *U*_min_ the harmonic energy penalty for the *m*th polymorph 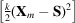 must be taken into account. *U*_min_ and **X**_min_ are elements of subsets of {**X**_1_, **X**_2_, …, **X**_*M*_} and {**H**_1_, **H**_2_, … **H**_*M*_} under the scope of a set value for **S**. Thus, 
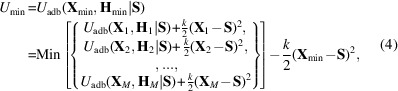
where *U*_adb_(**X**_*m*_, **H**_*m*_|**S**) represents the unbiased energy component of the *m*th polymorph generated adiabatically by using the biased PES *U*(**X**, **H**|**S**) and **X**_0_ = **S**. This strategy involving optimization of multiple polymorphs to obtain *U*_min_ as part of each EVCCP step is likened to the particle swarm approach (Kennedy & Eberhart, 1995 ▸) and also has similarities with umbrella sampling (Torrie & Valleau, 1977 ▸), as demonstrated schematically in Fig. 2 ▸.

**Figure 3:**
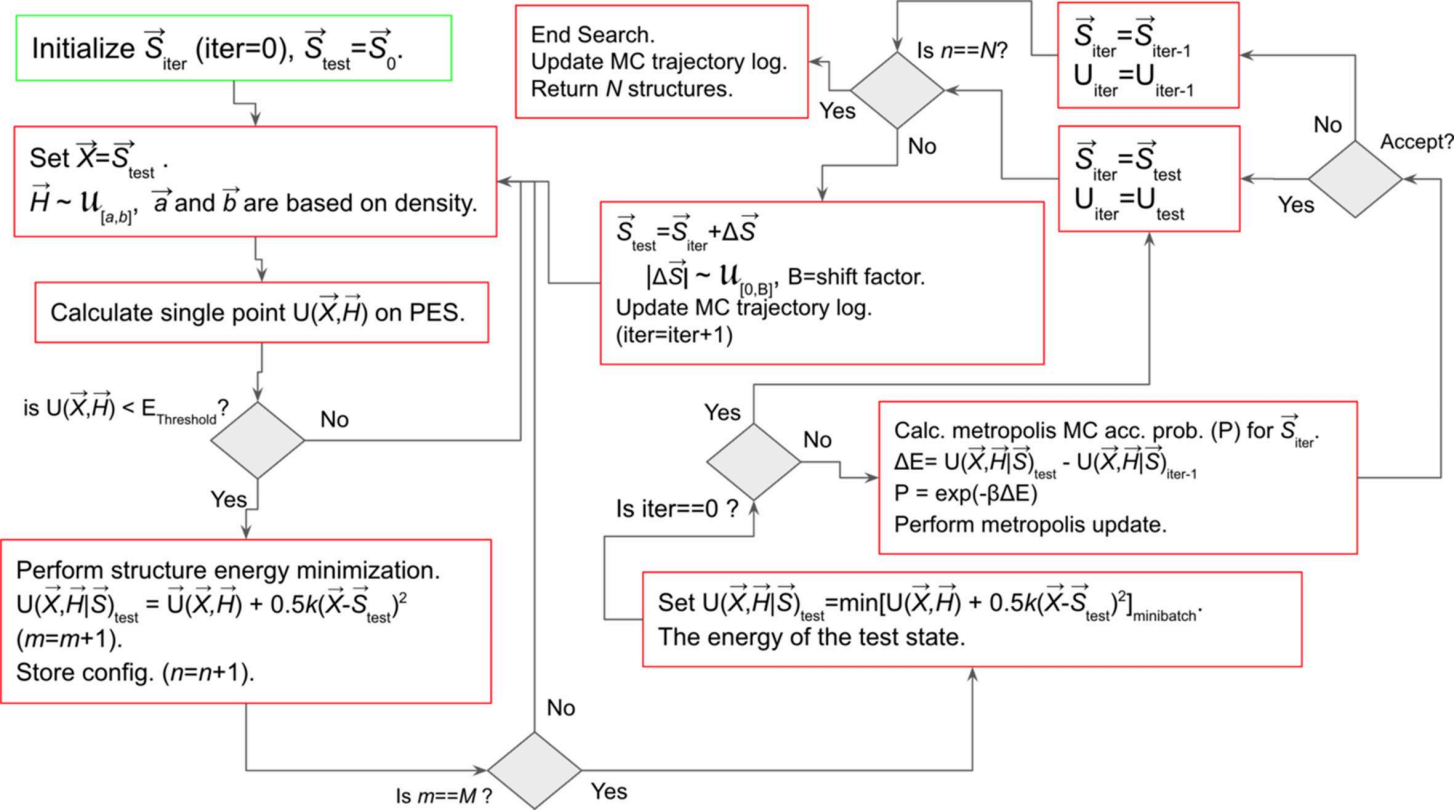
Flowchart of the EVCCPMC algorithm. In this schematic, the symbol ‘=’ represents the assignment operation, while ‘==’ is used for conditional evaluation.

A flowchart indicating the most relevant steps for coding of the EVCCPMC algorithm is shown as Fig. 3 ▸. Initialization of the algorithm requires **S**_0_ (iter = 0, *i.e.* 0th iteration) which is read from a previously known or randomly selected polymorph. This **S**_iter_ = **S**_0_ is input to the structure generator which is used to generate the mini-batch of *M* energy-minimized structures (see Fig. 2 ▸). It is the global minimum energy polymorph from the mini-batch that is used to obtain *U*(**X**, **H**|**S**_iter_). The next step is the metropolis update for **S**_iter+1_, *i.e.* generation of a test configuration **S**_test_ = **S**_iter_ + Δ**S**. A different batch of *M* structures are generated using **S**_test_ as the seed EV resulting in the test configuration energy *U*(**X**, **H**|**S**_test_) required for the update. This procedure is iterated (iter = iter+1) to determine each **S**_iter_ until *N* structures have been sampled. For the algorithm to work effectively, an *E*_threshold_ must be declared so that polymorph configurations with *U*(**X** = **S**_test_, **H**) > *E*_threshold_ can be rejected prior to the polymorph energy optimization because values for Δ**S** and **H** are generated randomly (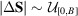 where *B* = *k*_B_*T*|Δ**S**|_max_) and can lead to unsuitable pre-optimization configurations. Each MC update for **S**_test_ is related to the corresponding estimate for *P*(**X**, **H**|**S**_test_) *i.e.* the polymorphs sampled by the polymorph generator for **S**_test_. Each step in the EVCCPMC trajectory contains both **S**_iter_, representing a node in a Markov chain of EV coordinates, as well as the collection of all *M* local minimum polymorphs.

## 3. Polymorph generation probabilities

When using PR or EVCCP sampling, the respective probabilities for polymorph generation *P*(**X**, **H**) or *P*(**X**, **H**|**S**) can be estimated using 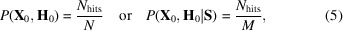
where *N*_hits_ is the total number of hits obtained for a specified polymorph (**X** = **X**_0_, **H** = **H**_0_), *N* is the total number of polymorphs generated for a PR search and *M* (the mini-batch size) is the number of polymorphs generated for a EVCCP step. Some benchmark for the dependence of these ratios on sampling sizes pertaining to this work will now be demonstrated for different *Z*′.

*P*(**X**_0_, **H**_0_|**S**) is further evaluated through histogramming and 2D-projection of *P*(**X**_0_, **H**_0_|**S** − **S**_0_). All sampling was performed in space group *P*2_1_2_1_2_1_ with *Z*′ = 1, 2 or 3 corresponding with the known coumarin polymorphs (*i.e.* form V, III and IV).

### 3.1. Sampling of probability distributions

**Figure 4:**
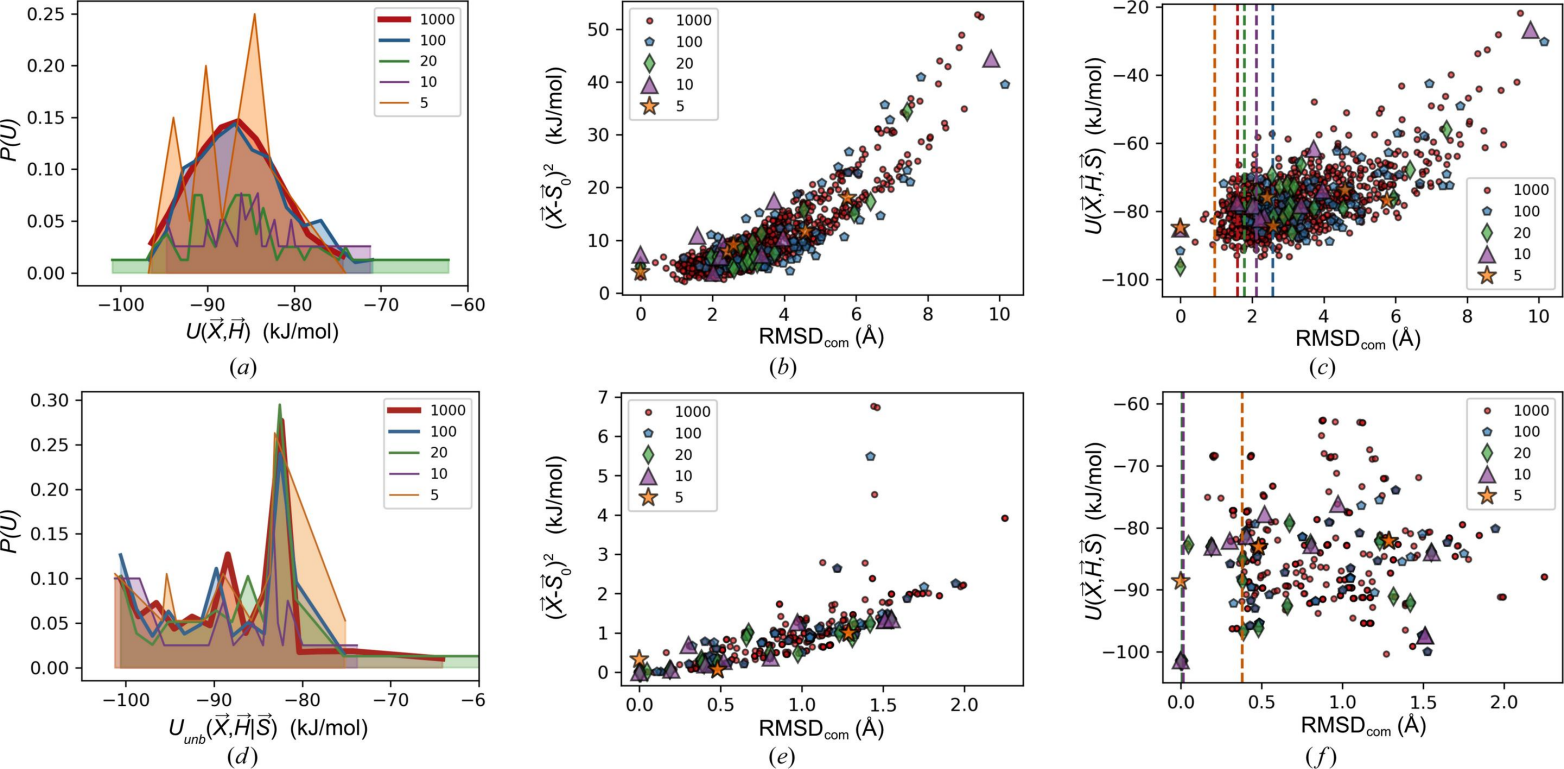
Plots for random (*a*)–(*c*) and EVCCP (*d*)–(*f*) coumarin *Z*′ = 3 polymorph data generated from searches. A different marker and color was used to differentiate the corresponding sample size (*N*, *M*) as indicated in the legend. The **S**_0_ coordinate is that of form IV. (*a*) and (*d*) compare *P*[*U*(**X**, **H**)] and *P*[*U*_unb_(**X**, **H**|**S**)] histograms; (*b*) and (*e*) compare plots of RMSD_com_ (*i.e.* a measure of the variance associated with an identified minima sampled) against the biasing penalty of *U*(**X**, **H**|**S**_0_); (*c*) and (*f*) are RMSD_com_ versus *U*(**X**, **H**, **S**) [see equation (2) and main text for explicit details].

Fig. 4 ▸ are the results from PR [Figs. 4 ▸(*a*)–4 ▸(*c*)] and EVCCP [Figs. 4 ▸(*d*)–4 ▸(*f*)] sampling of the set of *N*, *M* = {5, 10, 20, 100, 1000} for *Z*′ = 3 (results for *Z*′ = 1, 2 are made available in the supporting information). For this part of the study only a gentle coupling (*k* = 2) was applied for EVCCP such that effects are mostly resulting from setting **X** = **S**. Figs. 4 ▸(*a*) and 4 ▸(*d*) compare the overall post-sampling unbiased optimized energy distributions *P*[*U*(**X**, **H**)] which for EVCCP results are denoted as *P*[*U*_unb_(**X**, **H**|**S**)]. As expected, the estimate of these distribution functions becomes smoother as sample size (*N*) increases. For pseudo-random searches the *P*[*U*(**X**, **H**)] distribution appears more characteristic of the Maxwell Boltzmann distribution, the strong departure from this as shown in EVCCP sampling demonstrates the expected key differences between *P*(**X**, **H**) and *P*(**X**, **H**|**S**). In general, *P*[*U*_unb_(**X**, **H**|**S**)] is bimodal exhibiting peaks roughly situated at 〈*U*(**X**, **H**)〉 as well as *U*(**X**_0_, **H**_0_).

Figs. 4 ▸(*b*), 4 ▸(*c*) and 4 ▸(*e*), 4 ▸(*f*) compare the polymorph sampling using a measure of the variance of the molecular center components in **X** from those components corresponding with the minimum energy polymorph that was identified in that particular sampling distribution [*i.e.***X**_min_ from *U*_min_ as shown in equation (4)]. The variance measure for molecular centers (RMSD_com_) is thus defined as, 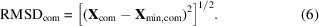
That is, polymorphs with RMSD_com_ = 0 represent the global minimum polymorph identified in a distribution. In Figs. 4 ▸(*b*) and 4 ▸(*e*) the RMSD_com_ is plotted against the harmonic biasing penalty term in equation (2) 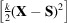. As expected, the value of this bias (*k* = 2) increases with the RMSD_com_. Also, the overall magnitudes of RMSD_com_ are much lower for EVCCP generated polymorph distributions.

Figs. 4 ▸(*c*) and 4 ▸(*f*) are plots of RMSD_com_ against the polymorph energy *U*(**X**, **H**, **S**) from equation (2) so that each polymorph energy includes the biasing contribution relative to **S**_0_ irrespective of how it was generated. Vertical dashed lines represent when the **X** was set to be **S_0_** for coumarin form IV. Figs. 4 ▸(*c*) and 4 ▸(*f*) are the proof of the important fact that RMSD_com_ = [(**S**_0, com_ − **X**_min, com_)^2^]^1/2^ can be seen to approach zero when sampling from a *P*(**X**, **H**|**S**) distribution as opposed to sampling from *P*(**X**, **H**).

The plots in Fig. 4 ▸ demonstrate the effect of increasing accuracy for *P*(**X**, **H**) or *P*(**X**, **H**|**S**) as sample sizes *N*, *M* → ∞. The comparison of Figs. 4 ▸(*c*) with 4(*f*) suggests that *M* can be small, in this case 5 < *M* < 10, so that **X**_min_ = **S**_0_.

**Figure 5:**
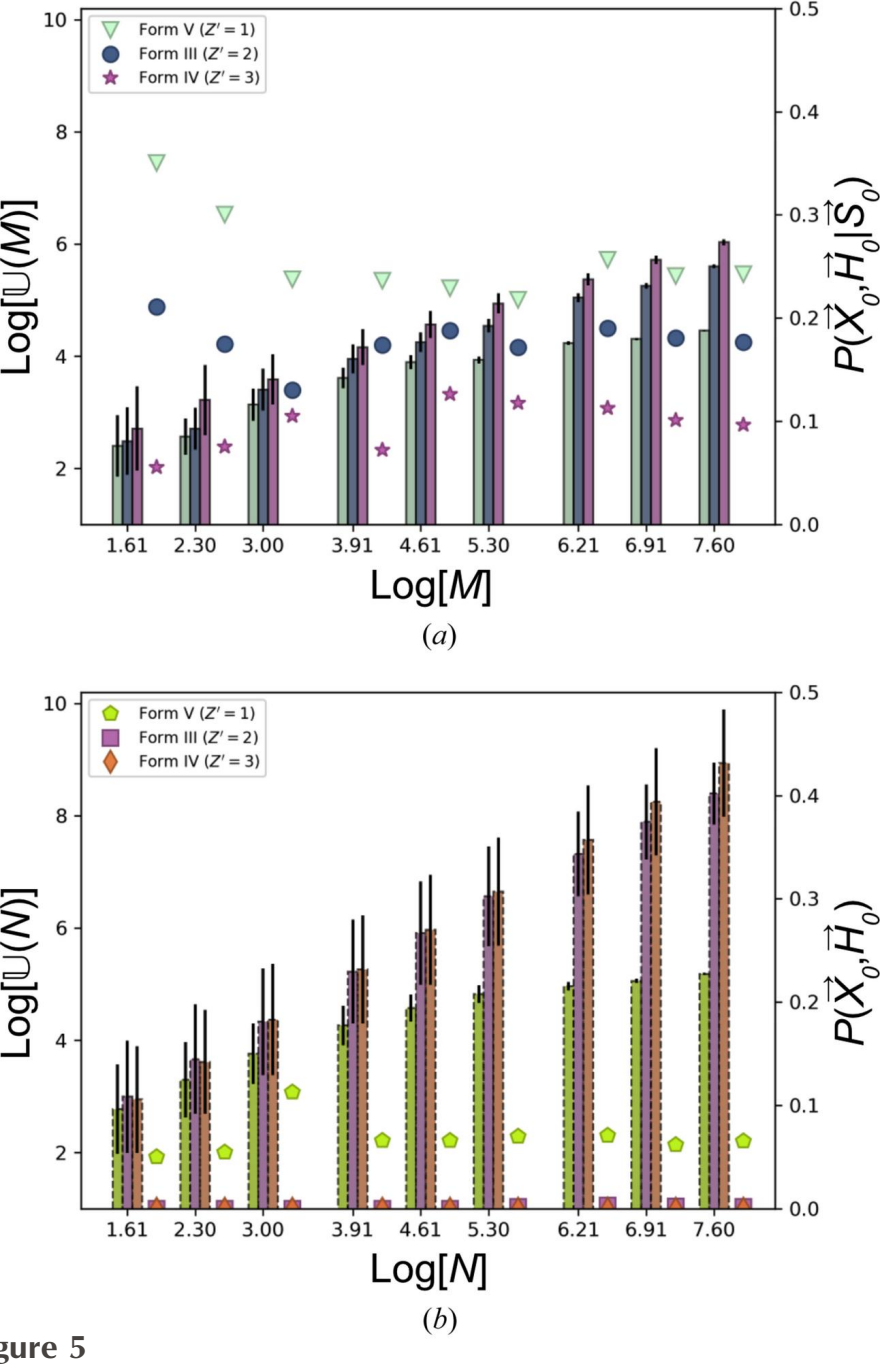
Log plots for the number of unique structures 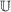 as a function of the number of polymorphs generated (*M* or *N*) from the respective EVCCP (*a*) or pseudo-random (*b*) search data. Bars are stacked as triplets for *Z*′ = 1, 2, 3 plotting 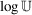 versus log(*N*) with the ratio 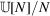 as error bars. In addition, different markers on the right of each triplet stack compare the conditional probability *P*(**X**_0_, **H**_0_|**S**_0_) to generate a specific polymorph from EVCCP (*a*) with the *P*(**X**_0_, **H**_0_) from a pseudo-random search (*b*). The scale for 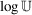 is on the left of each plot with the scale for probabilities on the right.

Raw MC generated polymorph data is commonly removed of duplicate configurations prior to stability ranking. The number of distinct or unique structures is denoted as 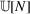. Fig. 5 ▸ shows bar plots of 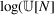 versus log(*N*) for the EVCCP searches [Fig. 5 ▸(*a*)] with 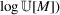 versus log(*M*) for PR searches [Fig.  ▸5(*b*)]. *N*, *M* values were ranged between [5, 2000]. Bars are stacked as triplets representing *Z*′ = 1, 2, 3 from left to right. The error bars show the ratio 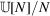 or 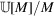. Markers on the right of each triplet are the corresponding *P*(**X**_0_, **H**_0_|**S**_0_) or *P*(**X**_0_, **H**_0_) estimate for each set of data [see equation (5)]. The center of each triplet is the log(*N*) or log(*M*) value for that group. As expected, the results demonstrate that 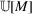 and 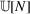 increase with *Z*′. Also when *M* = *N*, 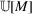 is lower than 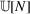 and 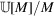 converges to small values with increasing *M* indicating that EVCCP does generate less unique configurations than a random search (the only exception is the case of *Z*′ = 1 PR search). *P*(**X**_0_, **H**_0_|**S**_0_) estimates are higher than *P*(**X**_0_, **H**_0_) and in fact *P*(**X**_0_, **H**_0_) is negligible for *Z*′ > 1 which assists to confirm **S** will bias sampling for a specific polymorph.

### 3.2. Sampling of polymorph conditional probability distributions and effect of coupling (*k*) magnitude

**Figure 6:**
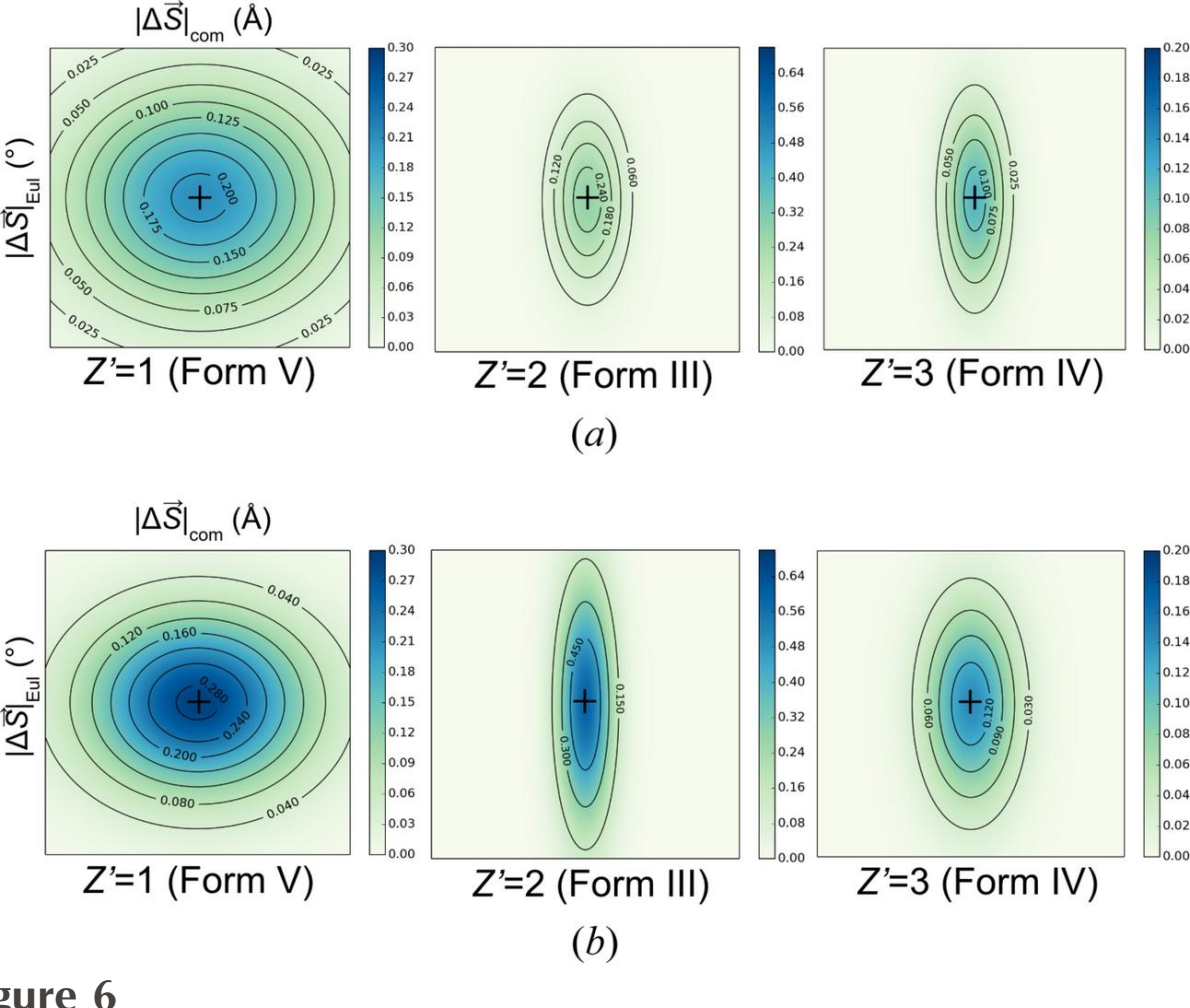
Plots of the bi-variate Gaussian fits to Table 1 ▸ data depicted as *P*(**X**_0_, **H**_0_|**S** − **S**_0_) for coumarin polymorphs with spring constants (*a*) *k* = 0 and (*b*) *k* = 1000. A black cross is positioned at |Δ**S**| = 0 for each particular form.

To demonstrate the *P*(**X**, **H**|**S**) underlying EVCCP, the related probability distribution *P*(**X**_0_, **H**_0_|**S**_0_ + Δ**S**) was estimated from sampling [see equation (5)] with a specific deviation (Δ**S** where 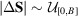 and *B* = |Δ**S**|_max_) from **S**_0_ (*i.e.***S**_0_ + Δ**S** = **S**). It is assumed that *P*(**X**_0_, **H**_0_|**S**_0_) will be highest (*i.e.B* = 0), with *P*(**X**_0_, **H**_0_|**S**) → 0 as 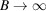. Differences in the curvature of *P*(**X**_0_, **H**_0_|**S** − **S**_0_) distributions for the *Z*′ = 1, 2, 3 example forms are mapped as 2D-projections (*i.e.* multi-variate → bi-variate) as the 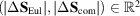 coordinate space (shown in Fig. 6 ▸) using the integer values for [*P*(**X**_0_, **H**_0_|**S** − **S**_0_)]^−1^ provided in Table 1 ▸. The values shown in Table 1 ▸ are directly related to the EVCCP parameter (*M*) required to ensure the specific polymorph can be generated. Clearly, the projections becomes narrower as *Z*′ increases. The reasonable fit of Table 1 ▸ values using a bi-variate Gaussian function demonstrates the suitability of the Gaussian approximation used for evaluating *U*(**X**, **H**|**S**) [see equation (3), and equations (32) and (33) in Appendix *A*[App appa]].

The expected effect of increasing the spring constant magnitude (*k*) on *P*(**X**, **H**|**S**) distribution is an increased probability at the origin and an overall narrower distribution. This is also shown in the sampling and the comparison between *k* = 0 and *k* = 1000 is shown in Table 1 ▸ and Fig. 6 ▸ for all *Z*′ examples.

**Figure 7:**
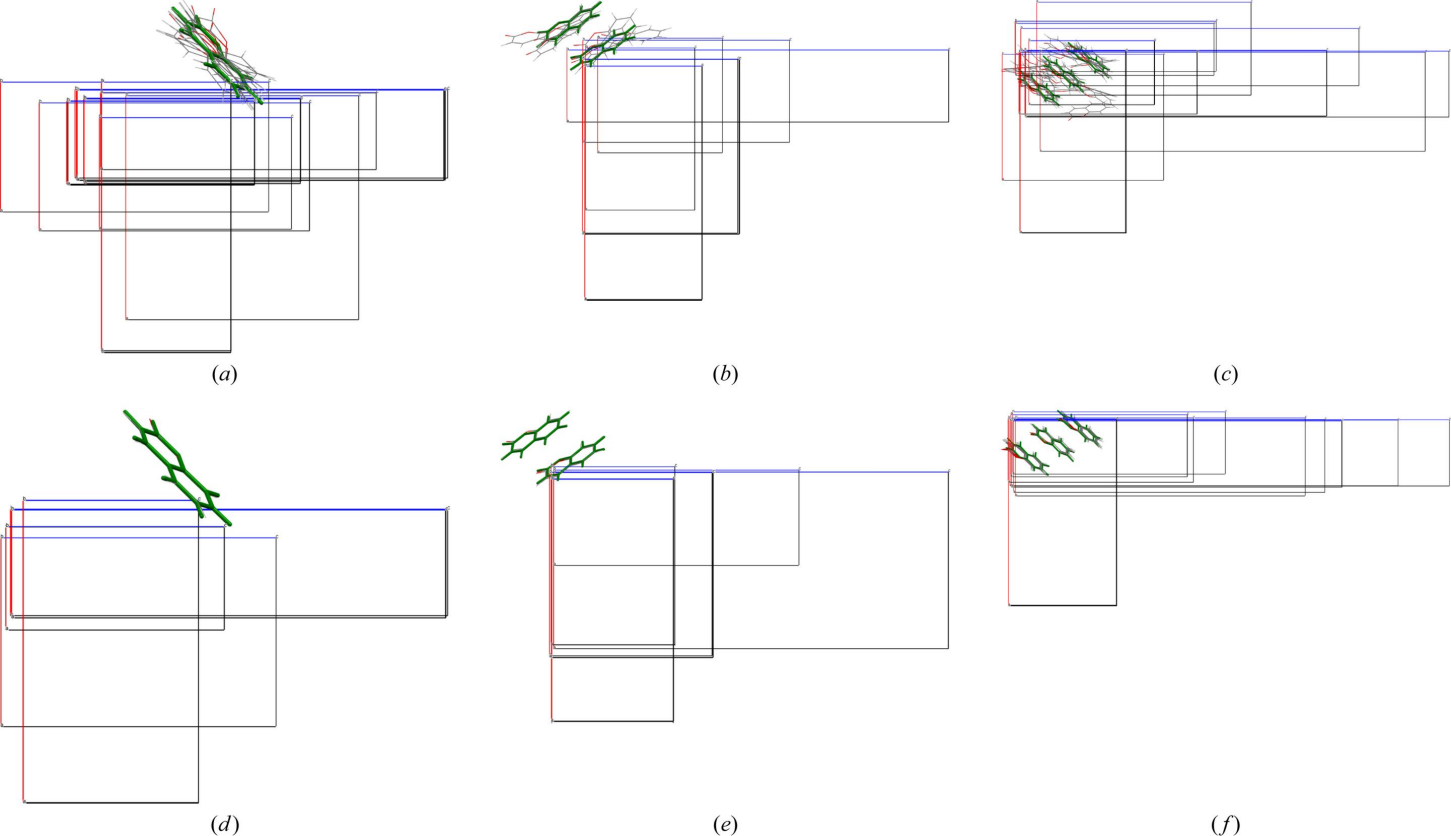
Visual representation of the effect of magnitude of harmonic coupling (*k*) during EVCCP sampling using different *Z*′. Displayed are the asymmetric units and unit cells projected down the *b*-axis as a multi-structure overlay between EVs (molecules with green outline) and corresponding 20 polymorphs generated (gray outline) for a single EVCCPMC step with a known coumarin polymorph for the reference EV. (*a*),(*d*) are *Z*′ = 1 form V with (*b*),(*e*) *Z*′ = 2 form III and (*c*),(*f*) *Z*′ = 3 form IV. The effect of weak coupling *k* = 10 [(*a*)–(*c*)] versus strong *k* = 1000 [(*d*)–(*f*)] is remarkable and at the heart of understanding the concept behind EVCCPMC.

The actual effect of *k* on polymorph generation each EVCCP step is best appreciated using Fig. 7 ▸. In Fig. 7 ▸ the reference EV are the same as in Table 1 ▸ only that *M* = 20 polymorphs are generated with either *k* = 10 or *k* = 1000. The resulting structures are arranged as a multi-structure projection down b with the origin as the center of the asymmetric unit. The difference between weak versus strong coupling can be clearly seen in that strong coupling results in fewer variants of polymorphs and that displacement from the reference system coordinates (molecules with green outline) between polymorphs is negligible.

## 4. Polymorph sampling with EVCCP

The EVCCP sampling threads were modified to enable replica exchange updates, following established principles in the field (Tuckerman, 2010 ▸; Frenkel & Smit, 2002 ▸). In this modified replica exchange (EVCCPMRE) approach, only the vector **S** traverses between replicas with different temperatures (*T*). This modification carries a significant implication: the configurational extended variables within **S** can encompass non-ordinal variables that influence structural energy and polymorph generation but remain invariant with respect to other components of **S** during the update moves within each *T* bath. In other words, these CSP search variables may lack obvious analytical derivatives, such as those linked to space group symmetry or *Z*′. A thorough evaluation of EVCCPMRE involving exchanges of variables such as space group is outside the scope of this investigation. Thus, the PES sampled in this report will be based on the marginal probabilities, given that variables like space group and *Z*′ are held constant in each MC run.

**Figure 8:**
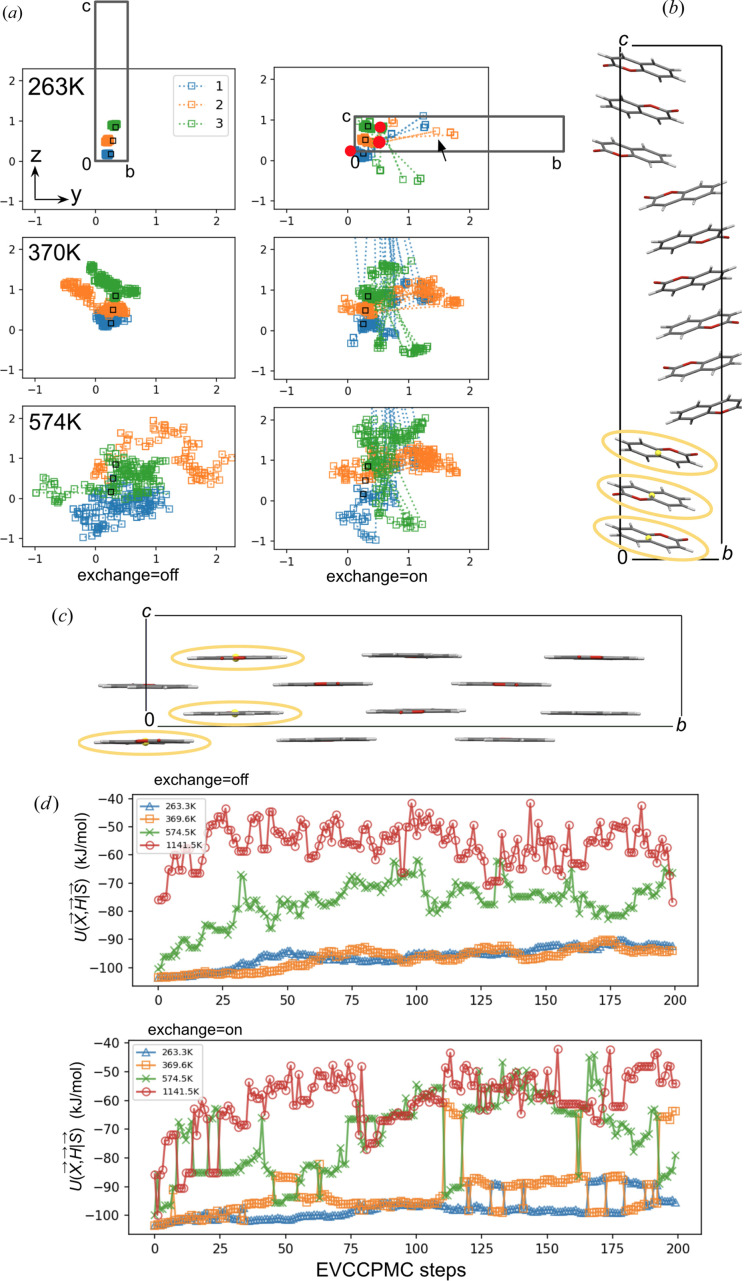
Comparison between EVCCPMC(exchange = off) and EVCCPMRE(exchange = on) for 263 K, 370 K, 574 K and 1142 K *T* bath 200-step trajectories with *Z*′ = 3 in space group *P*2_1_2_1_2_1_. (*a*) Molecular center components of **S** (molecules 1, 2 and 3) projected down reference system *x*-axis. Initial positions are highlighted with black squares. The black arrow indicates a configuration exchange event between baths. The last position in the EVCCPMRE 370 K bath is indicated with red circles. Overlays of pre-optimized unit cells for *U*_min_ polymorphs that were generated in the 100 K bath final step are shown with corresponding image of post-optimization structure for (*b*) MC and (*c*) MRE with yellow ovals outlining the molecules of the asymmetric unit. (*d*) The stepwise configuration energy *U*(**X**, **H**|**S**) is plotted for each bath.

Fig. 8 ▸ demonstrates how **S**_com_ fluctuate and diffuse about some local minima with a variance relative to *T*.

Tests of polymorph screenings using EVCCPMRE with coupling *k* = 1000 were performed for 13 space groups for both *Z*′ = 3, 4 and compared with analogous unbiased PR searches (*N* = 8000). Unless otherwise specified the initial configuration for seeding the MRE **S**_0_ was generated using a smaller preliminary random search (*N* = 100). Bath *T* were chosen based on the condition that the acceptance rate for exchange moves should be close to 0.5. Baths were set at 263 K, 370 K, 574 K and 1142 K (roughly exponentially spaced). Since commonly *P*(**X**_0_,**H**_0_) < [8000]^−1^, each search is not considered exhaustive, thus identifying the same global minima during comparison is not a requirement for enhanced sampling. In principle, the high *T* bath acts to scan for new global minima using large displacements in EV space whereas the lower *T* baths can harvest information about polymorphs in surrounding super basins (Yang & Day, 2021*a* ▸).

In Fig. 8 ▸(*a*) the molecular center EVs are plotted in projection down the *x*-axis of the reference system with the initial positions for **S**_com, 0_ indicated using black squares. Fig. 8 ▸(*d*) plots energy output *U*(**X**, **H**|**S**) stepwise from a test simulation. The trajectories are from simulation with 200 steps of MC (no replica exchange) compared with MRE. The trajectory data shown for Fig. 8 ▸ was for *Z*′ = 3 searches in space group *P*2_1_2_1_2_1_ starting with a predetermined global minimum polymorph that has *U*(**X**, **H**) = 103.8 kJ mol^−1^ (iso-structural with coumarin form IV). In the 370 K MC run, the final EV position for **S**_com_ regenerates the initial polymorph [shown in Fig. 8 ▸(*b*)], whereas in the 370 K MRE bath this is not the case, the resulting structure is shown in Fig. 8 ▸(*c*) with overlay of the unit cell and EVs (red circles) in Fig. 8 ▸(*a*). The effect of MRE is clear upon inspection of Fig. 8 ▸(*a*) with an example indicated using a black arrow. In contrast when exchange is disabled, EVs in comparative reference system do not have same degree of spacial coverage in the same number of MC steps.

### 4.1. EVCCPMRE variant schemes

EVCCPMRE was implemented using the Python interpreter as wrapper code to drive a modified version of the codes for the crystal structure generator *UPACK* (van Eijck & Kroon, 1999 ▸)[App appb]. Variations of EVCCPMRE were tested in order to identify if certain modifications of the workflow could further enhance the sampling and subsequent screening results. Despite the utilization of *UPACK* code for this work, these concepts are transferable and can be coded using many other publicly available crystal structure generators. The basic MRE algorithm as previously described is referred to as MRE0 (baths = 4, *k* = 1000, steps = 200, cycles = 1, *M* = 10, *N* = 8000).

A variant algorithm, MRE1, evaluates the addition of a history dependent biasing potential as a Gaussian kernel (Laio & Parrinello, 2002 ▸) placed along the EV path in attempts to further enhance sampling. In the MRE1 scheme, the historical biasing potential adds an energetic penalty to *U*(**X**, **H**|**S**) if the EV test-state **S** had already been previously visited.

The MRE2 scheme incorporates an additional forced update move which is referred to as ‘forced-relaxation’. The forced-relaxation move causes the system replicas to reset the EV state of each *T* bath, thus descend back into a local basin that was previously detected ‘on-the-fly’. The update occurs at the start of each MC cycle after a set number of MC steps. MRE2 is based on a notion that the EVs re-visit a low-energy basin after some predetermined length along the sampling path trajectory. When running MRE2 the mini-batch size was reduced (*M* = 5) so that twice as many MC steps will be run (baths = 4, steps = 40, cycles = 10, *M* = 5, *N* = 8000).

The final modification, MRE3 tests the performance of a global forced-relaxation resetting which occurs after several MC-cycles during the overall search (termed ‘relaxation-restart’). The MRE3 is similar to an MRE2 update, yet differs in that all polymorphs over a number of cycles from all replicas are evaluated for the unbiased [*U*(**X**, **H**)] global minimum required for restarting the MRE. This takes longer to run because the basic MRE0 does not perform structure optimizations on-the-fly to absolute full convergence each MC step (full convergence occurs during post-processing), but also differs because EVs for structures based on *U*(**X**, **H**|**S**) will be affected by the harmonic coupling *k*.

### 4.2. EVCCPMRE efficiency and comparison with pseudo-random search method

To facilitate a stepwise comparison of the sampling performance between variant searches, we devised a representative metric for the number density of unique configurations identified within a low-energy window. This was loosely based on earlier work with replica exchange ergodic metrics (Thirumalai *et al.*, 1989 ▸; Whitfield *et al.*, 2002 ▸). The choice was an average energy for the window of 20 lowest energy ranked structures made relative by using a mean-shifted value (*i.e.* 〈*U*〉_*T*20_ − 〈*U*〉). Monitoring of the relative 〈*U*〉_*T*20_ − 〈*U*〉 parameter was calculated stepwise from each set of search statistics as a function of number of polymorphs generated (*N*, corresponding with the number of MC steps). Evaluation of overall search performances was made using typical energy versus density landscapes from unbiased structure energy optimization of resultant distinct polymorphs. The notation *E* = *U*(**X**, **H**) is used for the unbiased polymorph energy and *E*_min_, 〈*E*〉_*T*20_ and percentiles of *E* (0.05%, 1.0%, 5.0%) were also evaluated.

**Figure 9:**
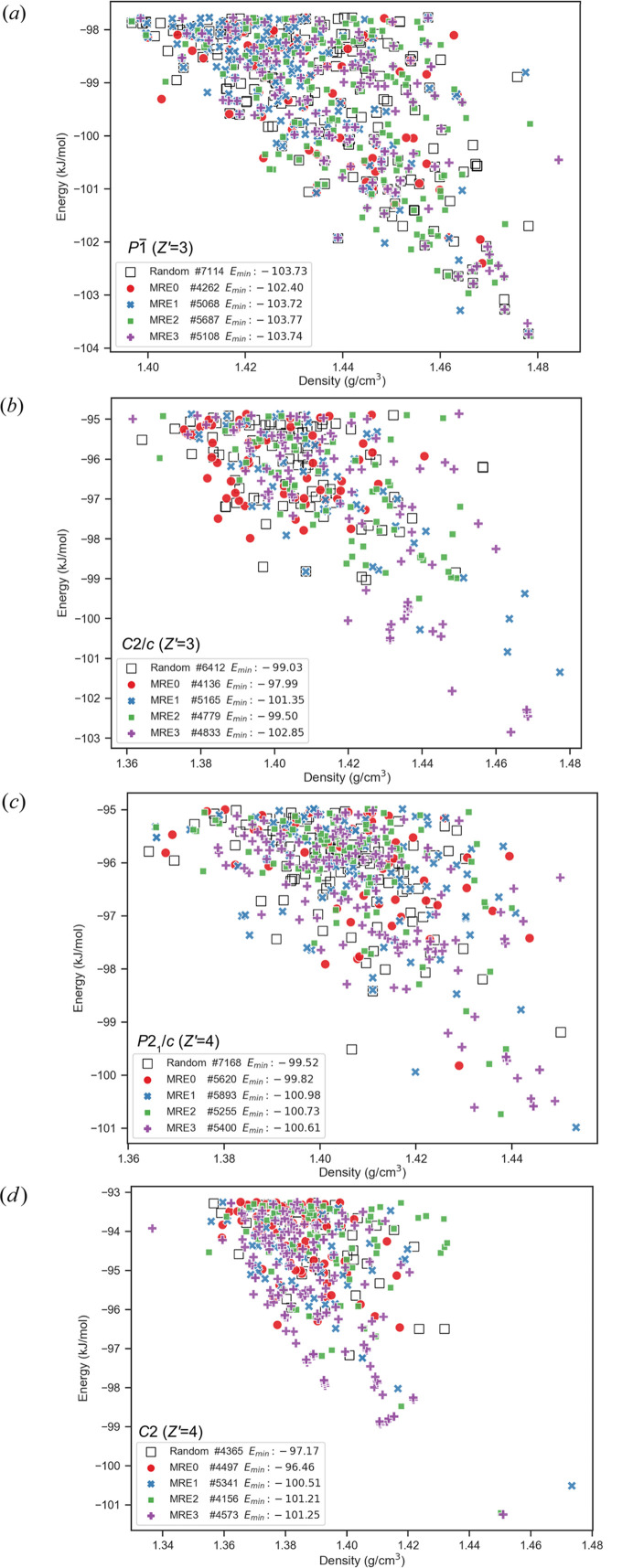
Example sets of polymorph energy versus density landscape plots resulting from the *N* = 8000 searches with EVCCPMRE (*k* = 1000) variant searches as differently colored markers and pseudo-random search results as the square black outlines. (*a*) and (*b*) are examples from *Z*′ = 3 with (*c*) and (*d*) from *Z*′ = 4.

**Figure 10:**
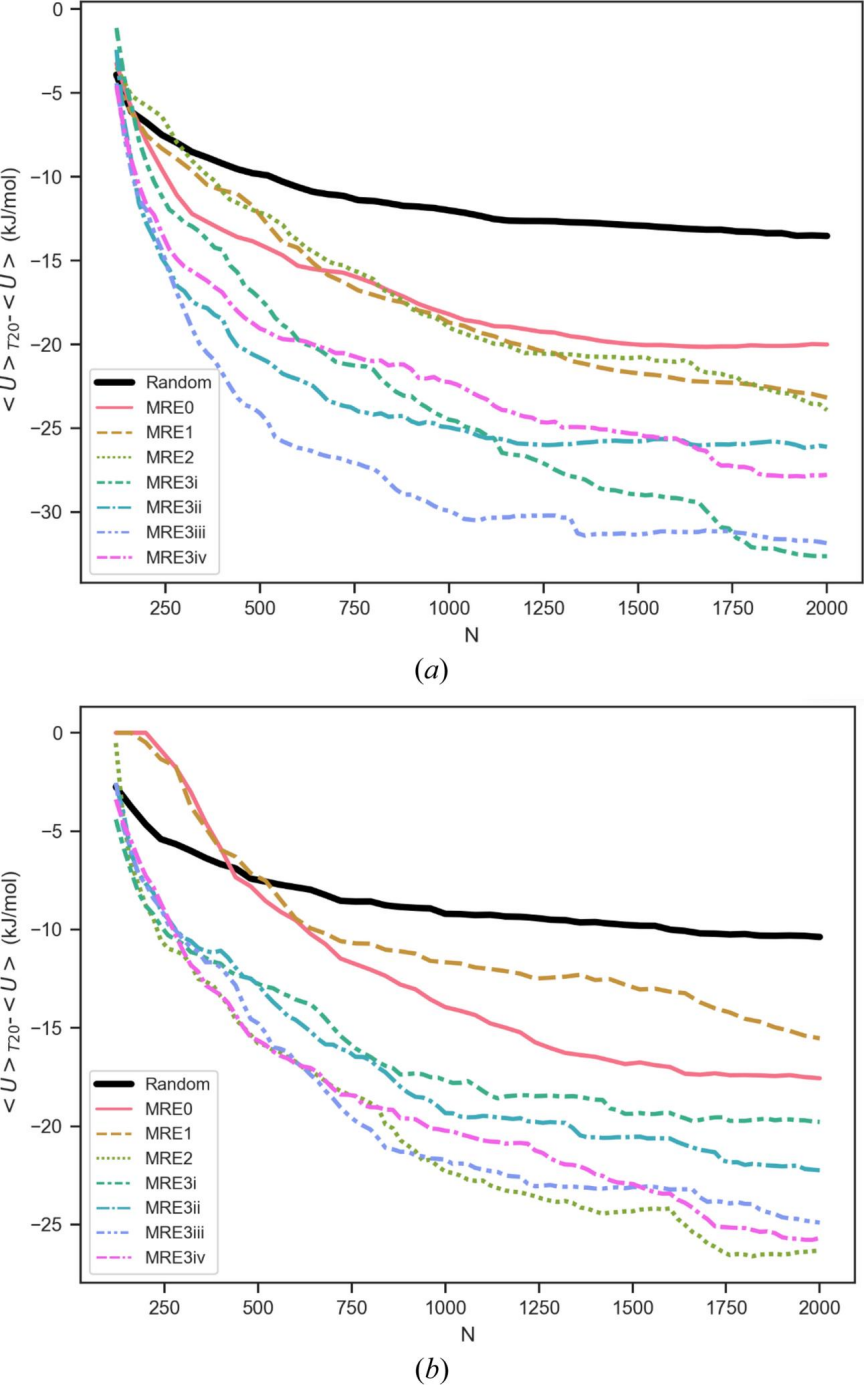
Example plots for search performance metric 〈*U*〉_*T*20_-〈*U*〉 plotted as a functions of the number of structures generated from *N* = 0 → 2000. Values from PR search trajectories are shown as black lines. Colored line plots are for EVCCPMRE searches with individual MRE3 runs (labeled i–iv, due to relaxation-restart) also included for comparison. Corresponding with Fig. 9 ▸ (*a*) is *Z*′ = 3 in space group *C*2/*c* with (*b*) *Z*′ = 4 using space group *P*2_1_/*c*. The measures from MRE searches obtain lower values in fewer steps and are thus interpreted as achieving a more efficient exploration of lower energy configurations.

Comparative landscape plots for *Z*′ = 3 searches (space group 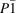 and *C*2/*c*) and *Z*′ = 4 searches (space groups *P*2_1_/*c* and *C2*) are shown as Fig. 9 ▸. Corresponding examples for the 〈*U*〉_*T*20_ − 〈*U*〉 metrics plotted as a function of *N* are shown in Fig. 10 ▸. The comparison of the 〈*U*〉_*T*20_ − 〈*U*〉 metrics in Fig. 10 ▸ demonstrates that EVCCPMRE sampling is more efficient at sampling low energy configurations. For variant searches the metric was able to reach the same value from the corresponding PS search in a smaller number of steps. For EVCCPMRE the metric appears to always converge to values lower than for PR. This does not imply that at this stage of development EVCCPMRE is more efficient at identifying new global minima (*E*_min_), since this would also be highly system dependant, rather that the EVCCPMRE sampling algorithm was behaving as intended, and the concept was correctly implemented.

Search metrics were evaluated over all space groups and summarized for larger intervals of *N* = 2000, 4000, 6000 and 8000 (see Table 2 ▸). In Table 2 ▸, the measures *R*_*g*_ and δ*E*_*g*_ are used to summarize comparisons over the many space groups. 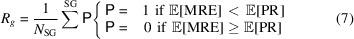
Here 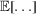 represents whether a 〈*U*〉_*T*20_ − 〈*U*〉 metric or *E*_min_ value is from MRE or PR sampling. The *R*_*g*_ value is the ratio between [0,1] for when 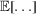 is less for MRE than for random sampling averaged over all the space group searches (*N*_SG_) for a particular *Z*′. If *R*_*g*_ > 0.5 it means that more MRE searches gave the lower 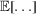 metric. 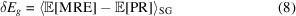
represents an average difference, over the space groups tested, between the 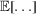 values being compared. The degree to which *E*_*g*_ < 0 represents the magnitude by which MRE searches obtained a lower 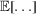 metric. From Table 2 ▸, the fact that values obtained δ*E*_*g*_ are slightly lower when running the modified MC updating schemes (algorithms MRE2 and MRE3) suggests that the forced-relaxation or relaxation-restart moves are useful options.

**Figure 11:**
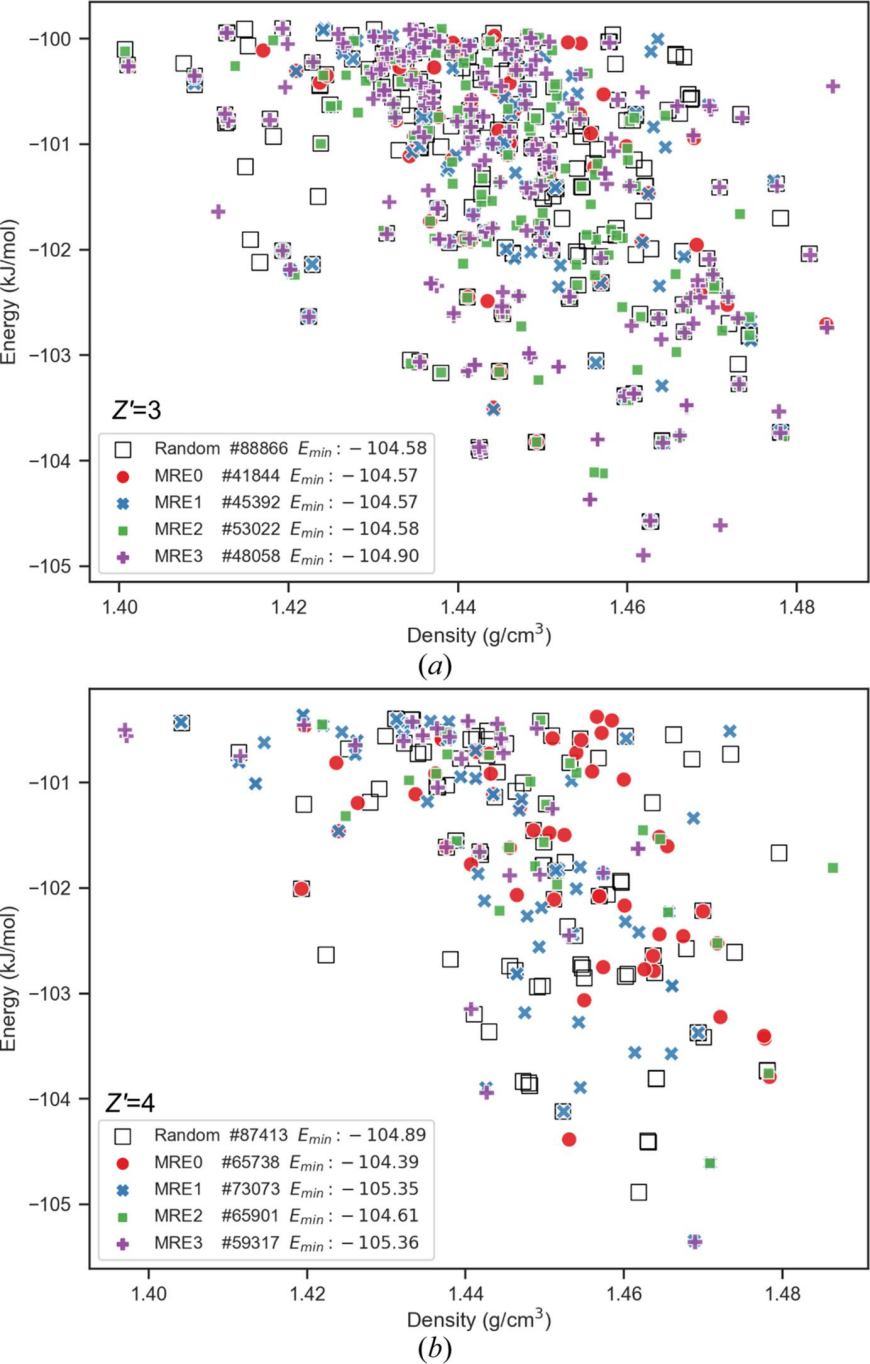
Low-energy high-density region from the crystal polymorph landscapes generated for (*a*) *Z*′ = 3 and (*b*) *Z*′ = 4 across all 13 space groups. Search results from EVCCPMRE variants are colored using different shaded markers with random searches as black square outlines.

Landscape plots that combine search data for all space groups are provided as Fig. 11 ▸, with corresponding *E*_min_, 〈*E*〉_*T*20_ values and percentiles provided in Table 3 ▸. Interestingly two of the *E*_min_ structures for *Z*′ = 4 were isostructural with the experimental coumarin form I polymorph (*Z*′ = 1, *Pca*2_1_) and represent the overall global minimum for the searches. It is likely a structure corresponding with experimental form II (*Z*′ = 2, *P*2_1_) was also generated.

Interestingly, the overall search results suggest any improvements in *E*_min_, 〈*E*〉_*T*20_ and percentiles made by EVCCP sampling were minimal or ill-defined. There was some expectation that enhancements may not be statistically significant (or highly system dependent) as these tests were restricted to a simple rigid body system and that *N* is too small (*i.e.* with limited MC steps the simulation EVs remain far from equilibrium behavior). In the limit of identical *N* in comparison, the overall coverage from EVCCPMRE was not expected to be as good as PR sampling due to the opportunistic exploration of a *k* biased search space which appears to sacrifice the total number of distinct hits. The results certainly re-demonstrate the robustness and acceptability of the PR and QR sampling strategies for CSP.

As expected the MRE searches do identify different polymorphs in low energy regions especially for the case of MRE2 and MRE3 for *Z*′ = 3. It is expected that many more might still be identified for large enough *N* such that the difference between the total number of distinct hits for MRE search versus PR method is reduced. Interpreting the results shown in Tables 2 ▸ and 3 ▸ is less useful for initial bench-marking or design of hyper-parameter defaults. It is very likely that EVCCP specific parameters such as *k* and Δ**S** might need further experimentation or on-the-fly adjustment especially when screening other molecular compounds with varying chemical complexity (*e.g.* molecules with many torsional degrees of freedom).

### 4.3. Benchmark for identification of rare polymorphs

Can EVCCPMRE increase the probability of generating a specific polymorph with a known intrinsically low probability of occurring from PR sampling? The candidate polymorph used for this part of our study was the experimentally identified form IV (*Z*′ = 3) of coumarin, which was previously reported to have a probability *P*(**X**_IV_, **H**_IV_) of 1 in 60000 occurring from PR searches (Shtukenberg *et al.*, 2017 ▸).

In order to make this evaluation, a *P*(**X**_IV_, **H**_IV_) estimate was made by running 20 (*Z*′ = 3) complete EVCCPMRE searches spawned with different initial CV coordinate (*N* = 8000) in space group *P*2_1_2_1_2_1_ and counting the number of times the form IV polymorph was generated. This was recalculated analogously using the PR method where it was found the probability was closer to 1 in 58000. The probabilities from variant EVCCPMRE search options are shown in Table 4 ▸. It is remarkable that the combination of both history-dependent biasing potential (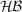) and forced-relaxation updates (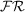) results in roughly a sixfold increase in the probability of successfully generating form IV. In hindsight, from the analysis of CSP landscapes (as documented in the previous section), any enhanced sampling effect from adding in the 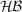 was less prominent if not spurious. However, for generating a specified rare polymorph, the utility appears more striking.

### 4.4. Free energy calculation

#### 4.4.1. Overview

Typically for studies in molecular simulation of crystal polymorphism, the temperature dependence of the free energy difference (FED) between individual forms is of considerable interest (Day *et al.*, 2003 ▸; Hoja *et al.*, 2017 ▸; Parrinello & Rahman, 1981 ▸; Reilly & Tkatchenko, 2013 ▸; Yu & Tuckerman, 2011 ▸). This is because a realistic ranking of polymorphs is attained by accurately factoring finite temperature effects. In practice, most methods involve direct calculation of phonon spectra to determine entropic contributions from a vibrational partition function. Strategies also exist which are based entirely on sampling with MD or MC finite temperature simulations. However, all methods are both approximate and computationally expensive (Baroni *et al.*, 2001 ▸; Martoňák *et al.*, 2003 ▸; Reilly & Tkatchenko, 2015 ▸; Nyman & Day, 2015 ▸; Frenkel & Ladd, 1984 ▸).

In an EVCCPMC simulation, the EV evolution includes contributions from the ensemble of crystal polymorphs to which there is the harmonic tether *k*. The EV are said to be scanning the polymorph probabilities at a finite temperature and it is assumed that thermodynamic ensemble averages such as free energy differences are derivable from a collective of EVCCPMC simulation trajectories. It is believed that the concept can be validated and such a FED estimate might be useful in future as a qualitative measure. To demonstrate, as example of such an ensemble FED estimate was determined, namely the FED between nominal variables *Z*′ = 1 and *Z*′ = 2. In this study we used the free energy perturbation (FEP) method for the FED calculation (Zwanzig, 1954 ▸). FEP from ECVCCP was appealing since it is straightforward to implement, requiring the EVs as input coordinates since a modified potential for *U*(**X**, **H**|**S**) could be evaluated (see Appendix *A*[App appa]).

#### 4.4.2. Free energy perturbation

The FED between *Z*′ = 1 and *Z*′ = 2 can be determined using EVCCPMC as the follows.

Given 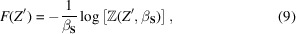
then 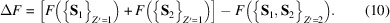
To do this a *Z*′ = 2 trajectory was generated at a specified *T* and the resulting EV coordinates are fed back into the polymorph generator with settings for *Z*′ = 1. Because a *Z*′ = 2 calculation will generate two sets of configurations the energy is re-weighted to account for stoichiometry.

The *U*(**X**, **H**|**S**) will be affected both by the harmonic coupling constant (*k*) and the displacement factor (Δ**S**) for the shift magnitude which generates MC test positions. For this work, multiple simulation runs were performed with a range of different Δ**S** and *k* depending on *T*. The deviation of MC acceptance/rejection (AR) ratio from 0.5 was used as a guide (Frenkel & Smit, 2002 ▸) to ensure simulation results were reasonable. All *Z*′ = 2 (*P*2_1_2_1_2_1_) runs start from a known pre-determined global minimum EV (**S**_*g*0_) corresponding with coumarin form III. Multiple trajectories (steps = 10000) were generated at twelve exponentially spaced *T* between 25 and 388 K. Spring constants ranged from 100 to 5000 in units of kJ mol^−1^ Å^−1^ for COM displacements or kJ mol^−1^ °^−1^ for Euler angles.

#### 4.4.3. Simulation results

As expected, it was found that at very low temperatures *U*_min_ values do not deviate much since the conditional probability *P*(**X**_*g*0_, **H**_*g*0_|**S**_*g*0_) was reasonably high (up to 0.33 with *k* = 1000 and *M* = 20). Also, the EV coordinates **S** fluctuate about the point **X**_*g*0_ typical of the behavior for a system tethered to a harmonic spring. As the temperatures are increased the biasing components of the energies were higher and EVs move away from **S**_*g*0_.

**Figure 12:**
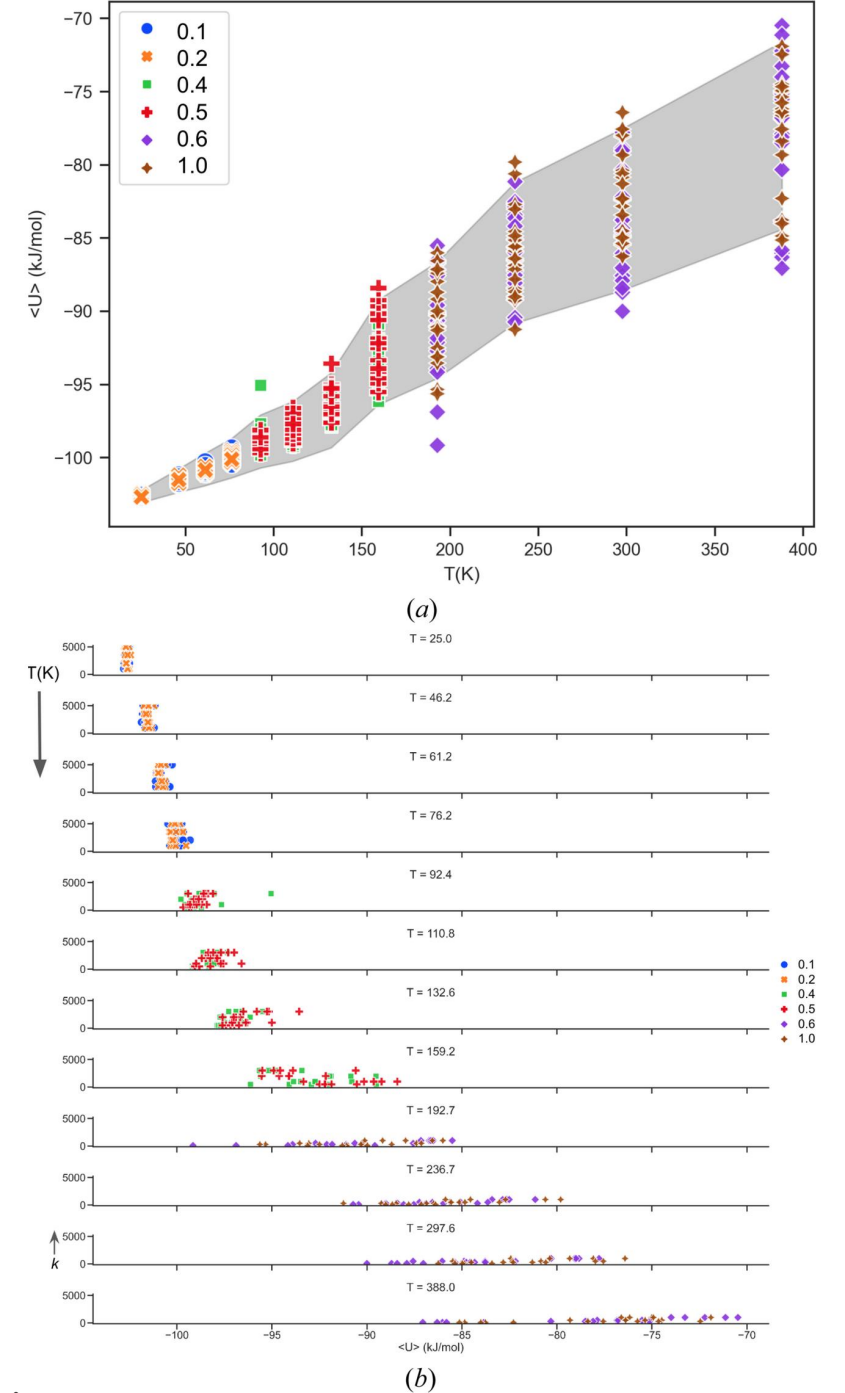
The 〈*U*〉 values from EVCCPMC simulations at various *T* are plotted with filled area (gray) representing the RMSD. (*a*) Colored markers for the data points represent the different values for |Δ**S**_com_| used. (*b*) Row plots of 〈*U*〉 are stacked for each *T*. The vertical axis on each subplot corresponds with constant *k* which takes on values 0 → 5000.

The plots of 〈*U*〉 are shown in Fig. 12 ▸. Each system was considered equilibrated after 2000 MC steps and 〈*U*〉 are averaged over the last 8000 steps.

**Figure 13:**
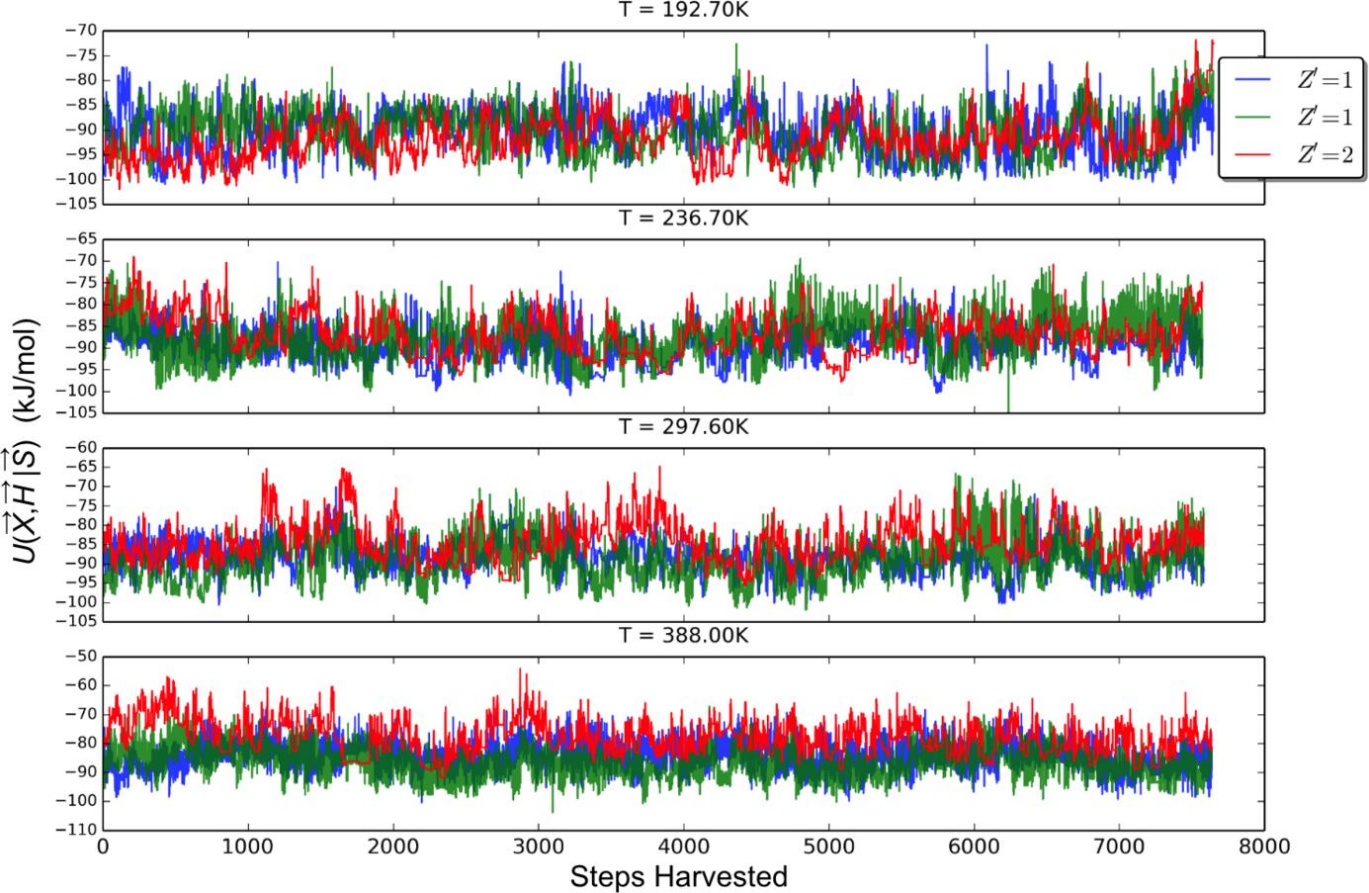
Instantaneous *U*(**X**, **H**|**S**) taken from EVCCPMC trajectories and used for FEP. The red signal trace is from the *Z*′ = 2 simulation. The green and blue traces are a re-sampling of *U* with *Z*′ = 1 using the EVs from the red trace.

**Figure 14:**
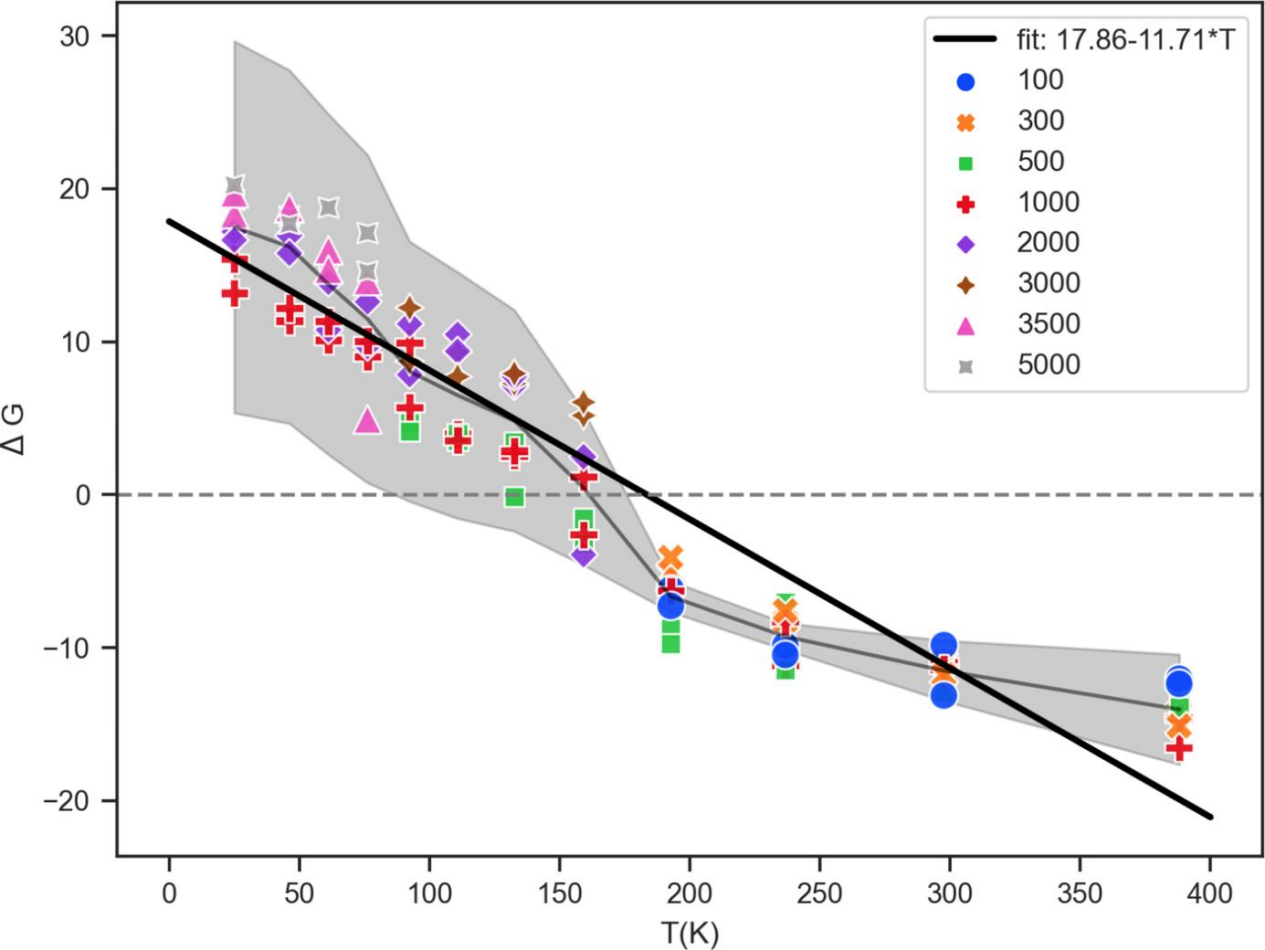
The 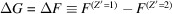 versus *T* linear relation [equation (13)] for coumarin polymorphs in space group *P*2_1_2_1_2_1_ fitted using data points from EVCCPMC simulations. Different markers represent *k*. The 〈Δ*G*〉 at each *T* is plotted with a dark gray line. The gray filled area is the magnitude |〈Δ*U*〉| centered at 〈Δ*G*〉.

The comparison of the instantaneous *U*(**X**, **H**|**S**) for a few different trajectories is depicted in Fig. 13 ▸. A plot of the FED values (with 〈Δ*U*〉 representative of the associated error) between *Z*′ = 2 and *Z*′ = 1 for coumarin crystal polymorph ensembles in space group *P*2_1_2_1_2_1_ is shown as Fig. 14 ▸. To facilitate the FED estimate, recall that a vibrational free energy curve (Nyman & Day, 2015 ▸) can be expressed as 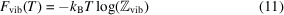
with 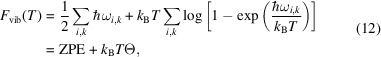
where ω_*i*, *k*_ are phonon frequencies. A Δ*F*_vib_(*T*) difference between any two such curves (*e.g.*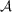 and 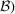 can then be approximated as 
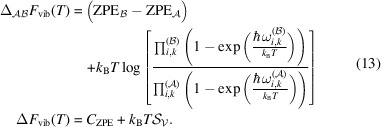


Thus the estimate for the FED between two curves can be grossly simplified as a straight line with a slope (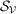). Despite being unrelated calculations and a high degree of error, there is a correspondence (albeit coincidental) with experimental observations and DFT based phonon calculations (Shtukenberg *et al.*, 2017 ▸). For coumarin space group *P*2_1_2_1_2_1_, experimental form III (*Z*′ = 2, *E*_0 K_ = −103.977 kJ mol^−1^) and form V (*Z*′ = 1, *E*_0 K_ = −102.366 kJ mol^−1^) have *E*_0 K_ values close to the global minimum configurations (*E*_min_). For fitting the 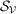 to the data points, the ZPE difference (*C*_ZPE_) was not negligible and is fit as an intercept which takes on a positive value indicating that *Z*′ = 2 structures are expected to be more favorable at lower temperatures. At higher temperatures a transition point at ≃ 200 K occurs. Above this transition temperature *Z*′ = 1 polymorphs in *P*2_1_2_1_2_1_ are predicted to be more favorable due to an entropic stabilization. This is in agreement with results identified using PBE(0)+MDB DFT-based phonon calculations that demonstrated form V should be significantly stabilized when harmonic vibrations and zero-point energies are taken into consideration (Shtukenberg *et al.*, 2017 ▸). However, suggesting that the answer to why one polymorph is more likely to crystallize than another may be qualitatively derived by ensemble averaging of many polymorphs is an overly bold statement which still remains invalid.

## 5. Conclusion

A new EV-based approach to CSP has been investigated. The approach relies on harmonic coupling between the reference EV coordinate system and the PR polymorph generator. Results of comparison of EVCCPMRE versus PR based on the coumarin system showed that EVCCP does not always lead to an overall greater yield of polymorphs in the low energy high density region of a landscape. It is believed this might be attributed to the selected Δ**S** and *k* used in the evaluation but is mostly attributed to the fact that coumarin CSP can be adequately performed within a rigid molecule approximation. It is believed that larger molecular systems with many more degrees of freedom (*i.e.* more rotatable bonds) would benefit from the EVCCPMRE approach in contrast to the PR method.

EVCCPMRE can be modified with history dependent biasing (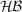), forced-relaxation (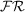) or relaxation-restart (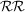) approaches to further enhance sampling. This was evidenced from the sampling statistics for coumarin form IV.

Averaging of 〈*U*〉 from EVCCPMC trajectories and performing FEP is one strategy to obtain a FED between ensembles of polymorphs (*i.e. Z*′ or space group). The FED tested was for the propensity of either *Z*′ = 2 or *Z*′ = 1 polymorphs to crystallize and by a sheer coincidence was found to correspond with the attributed entropic stabilization at *T* > 300 K that lead to the discovery of coumarin form V (*Z*′ = 1) from melting and cooling experiments.

## Supplementary Material

Sampling statistics of probability distributions comparing from pseudo-random and EVCCP methods for Z'=1 and Z'=2. Web links to all the codes related to this article. DOI: 10.1107/S205252062400132X/aw5082sup1.pdf

## Figures and Tables

**Figure 15:**
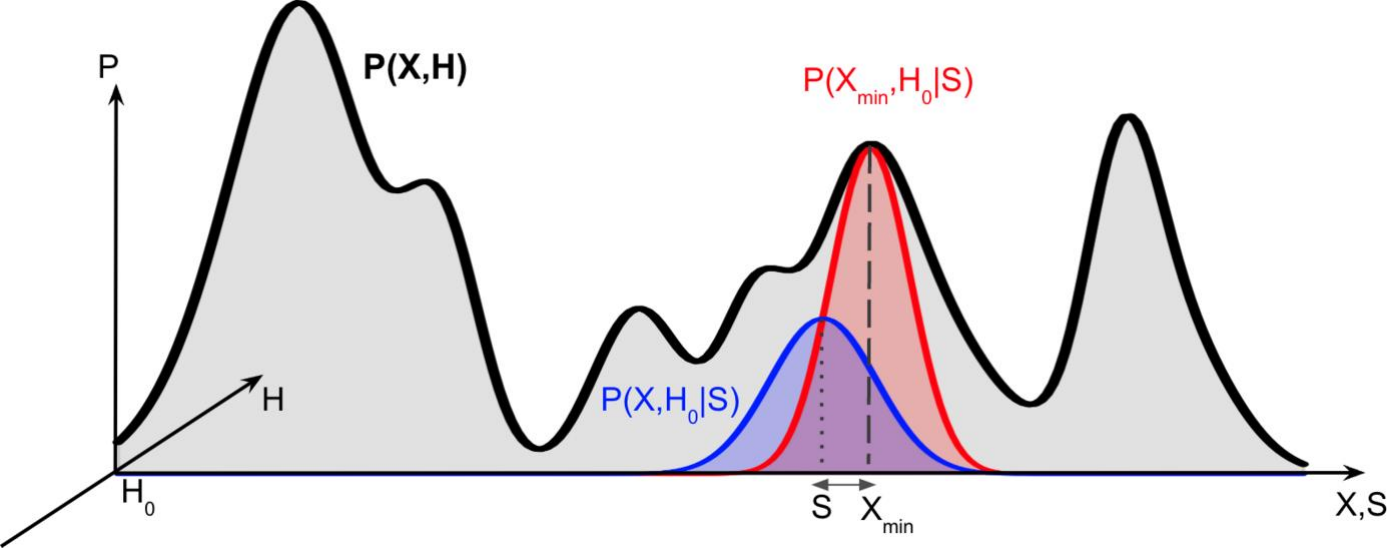
The EVCCP approach is based on an ideal relationship between conditional probability densities *P*(**X**, **H**|**S**) which are approximated as Gaussian and the actual probability of sampling any structure *P*(**X**, **H**). To help illustrate this idea a fictitious 1D plot of the probability distribution for *P*(**X**, **H**), which is difficult to estimate in practice, is projected down the **H**-axis located at **H**_0_. The red-shaded *P*(**X**_min_, **H**_0_|**S**) centered on **X**_min_ demonstrates how the probability of obtaining **X**_min_ will be highest when **S** = **X**_min_. The blue-shaded *P*(**X**, **H**_0_|**S**) is centered on **S** and has its width and height controlled by *k*. The blue distribution shows the actual probability for sampling of **X**_min_.

**Table:** 1(*a*), 1(*b*) and 1(*c*) are, respectively, *Z*′ = 1, 2, 3 for coumarin form V, III and IV (*k* = 0). Rows index the |Δ**S**|_max_ increments for molecular centers |Δ**S**_com_| (0.1–1.5 Å), with columns being increments for Euler angles |Δ**S**_Eul_| (5–50°). The effect of *k* = 1000 is shown as 1(*d*), 1(*e*) and 1(*f*).

Table 1(*a*)					
|Δ**S**|_max_	5	10	25	30	50
0.1	4	4	6	6	21
0.2	6	5	8	7	16
0.5	11	10	10	9	17
1.0	9	10	14	14	19
1.5	11	11	11	16	18

**Table:** 

Table 1(*b*)					
|Δ**S**|_max_	5	10	25	30	50
0.1	6	6	8	9	19
0.2	8	10	12	11	31
0.5	57	52	89	67	123
1.0	320	145	267	800	533
1.5	388	227	320	1600	800

**Table:** 

Table 1(*c*)					
|Δ**S**|_max_	5	10	25	30	50
0.1	11	12	13	18	267
0.2	27	21	39	43	200
0.5	178	89	200	267	–
1.0	800	526	–	–	–
1.5	–	–	1538	800	–

**Table:** 

Table 1(*d*)					
|Δ**S**|_max_	5	10	25	30	50
0.1	2	3	5	7	11
0.2	5	5	6	10	31
0.5	8	8	9	12	48
1.0	10	8	12	14	23
1.5	7	11	13	14	42

**Table:** 

Table 1(*e*)					
|Δ**S**|_max_	5	10	25	30	50
0.1	3	4	3	5	6
0.2	7	5	5	7	21
0.5	5	57	40	22	–
1.0	7	145	100	178	200
1.5	61	265	–	320	800

**Table:** 

Table 1(*f*)					
|Δ**S**|_max_	5	10	25	30	50
0.1	7	9	8	11	100
0.2	15	9	18	17	267
0.5	–	100	–	–	–
1.0	320	–	–	–	–
1.5	198	–	1600	784	–

**Table:** *R*_*g*_ and δ*E*_*g*_ are measures representing the degree the energy metric differs between MRE and pseudo-random searches. Table 1(*a*) lists 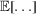 for *E*_min_ and Table 1(*b*) has 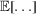 as the 〈*U*〉_*T*20_ − 〈*U*〉 metric.

2(*a*)										
			*R*_*g*_ @ *N* =				δ*E*_*g*_ @ *N* =			
*Z*′	Variant	*M*	2000	4000	6000	8000	2000	4000	6000	8000
3	MRE0	10	0.23	0.15	0.23	0.23	0.98	1.27	1.17	1.23
3	MRE1	10	0.46	0.38	0.38	0.31	0.27	0.61	0.61	0.60
3	MRE2	5	0.62	0.38	0.46	0.31	0.10	0.80	0.17	0.38
3	MRE3	5	0.46	0.46	0.38	0.46	0.27	0.37	0.41	0.13
4	MRE0	5	0.23	0.23	0.23	0.15	0.93	1.50	1.17	1.48
4	MRE1	5	0.38	0.38	0.46	0.23	0.81	1.63	1.40	1.82
4	MRE2	5	0.31	0.31	0.23	0.23	0.12	0.76	0.94	0.93
4	MRE3	5	0.31	0.15	0.23	0.46	0.45	1.63	0.87	0.75

**Table:** 

2(*b*)										
			*R*_*g*_ @ *N* =				δ*E*_*g*_ @ *N* =			
*Z*′	Variant	*M*	2000	4000	6000	8000	2000	4000	6000	8000
3	MRE0	10	0.92	1.00	1.00	1.00	−6.37	−7.57	−8.47	−9.14
3	MRE1	10	1.00	1.00	1.00	1.00	−6.84	−8.36	−9.85	−10.88
3	MRE2	5	1.00	1.00	1.00	1.00	−12.17	−14.17	−15.52	−16.32
3	MRE3	5	1.00	0.92	0.92	1.00	−10.91	−10.48	−10.65	−13.27
4	MRE0	5	0.92	1.00	1.00	1.00	−4.04	−4.74	−5.98	−7.08
4	MRE1	5	1.00	1.00	1.00	1.00	−4.33	−6.47	−7.68	−8.64
4	MRE2	5	0.92	0.92	0.92	0.92	−10.44	−12.93	−14.11	−14.83
4	MRE3	5	1.00	1.00	1.00	1.00	−10.03	−10.75	−10.10	−10.33

**Table 3:** Composite unbiased energy metrics [*E* = *U*(**X**, **H**) in kJ mol^−1^] evaluated over the multiple search types from the 13 space groups The latter three columns are percentiles of *E*.

Variant	*#*Hits	*E*_min_	〈*E*〉_*T*20_	*E*@0.05%	*E*@1%	*E*@5%
*Z*′ = 3						
Random	88866	−104.575	−103.577	−102.061	−97.802	−94.954
MRE0	41844	−104.566	−102.635	−101.485	−97.466	−94.707
MRE1	45392	−104.574	−102.760	−102.005	−97.712	−94.858
MRE2	53022	−104.579	−103.465	−102.637	−97.844	−94.854
MRE3	48058	−104.897	−103.987	−103.369	−98.597	−95.110
*Z*′ = 4						
Random	87413	−104.886	−103.598	−101.706	−96.486	−93.387
MRE0	65738	−104.385	−102.675	−101.110	−96.174	−93.269
MRE1	73073	−105.346	−103.260	−101.116	−96.308	−93.250
MRE2	65901	−104.611	−101.783	−100.188	−96.004	−93.309
MRE3	59317	−105.358	−101.647	−100.426	−96.541	−93.528

**Table 4:** Estimates of inverse probabilities for coumarin form IV generation using different *Z*′ = 3 searches in space group *P*2_1_2_1_2_1_ Search options: 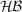 = History dependent biasing; 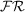 = Forced-relaxation updating.

Search	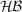	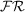	*P*(**X**_IV_, **H**_IV_)^−1^
Random	No	No	58000
EVCCPMRE	No	No	32000
EVCCPMRE	Yes	No	17778
EVCCPMRE	No	Yes	11428
EVCCPMRE	Yes	Yes	8421

## Footnotes

1 If β = 0 then this is the special case of *T* = ∞ condition equivalent with the random search method.

## Appendix A Theoretical framework

### A1. Theoretical description for an extended variable reference coupled to crystal polymorph configurations

Generally, a configurational partition function, as applied in molecular simulation, represents the phase space comprising all possible microstates in a system of *N* molecules (Tuckerman, 2010 ▸). The partition function is used to statistically derive thermodynamic properties. We will make a simplification and state that one subset of these microstates encompasses possible crystal polymorphs for a molecule. The configurational variables in this subset are those necessary for describing these polymorphs and can include hidden variable types representing both translational and point symmetry.

Let  represent this rudimentary configurational partition function of the crystal polymorph space for a molecular system, defined as follows:

Here  has components that are collective variables (CV), which are the positional coordinates for molecular centers (Å) as well as relative molecular orientations in Euler angles (°). In cases where rotations about a bond can occur **X** will also contain these angular coordinates. The number of components in **X** is *d* = *Z*′*6 + *n*_tor_ where *Z*′ is the number of molecules in the asymmetric unit and *n*_tor_ are the number of rotating bonds.  are the vector coordinates for a parallelepiped or (unit cell). **H** ≡ {**a**, **b**, **c**} where **a**, **b** and **c** are the unit-cell vectors in units of Å. It is worth mentioning that in general when  the system volume (*V*) and number of molecules (*N*) are fixed quantities which is not the case here. The states for the system are limited to involving only a certain fixed number of molecules (*Z*) that can occupy a parallelepiped (unit cell) having parameters denoted as **H** (treated as integration variables). Thus, *Z* is effectively replacing the *N* in the  formalism. For any such system *Z* is also related to the space group symmetry (SG) which is considered a hidden constraint variable, held constant, affecting the number of molecules in the asymmetric unit (*Z*′). For example,  would represent the configurational partition function for all *Z*′ = 2 structures in all possible space groups (there are 230 space groups, however most molecular crystals will commonly manifest in a smaller subset of < 30 of these space groups).  can then be further subdivided into subsets of  each with a fixed respective space group variable [*e.g.* = 2, SG = *P*2_1_/*c*)]. The 0 K potential energy surface (PES) for the system is represented with *U*(**X**, **H**) and is a rough multidimensional surface. *U*(**X**, **H**) is associated with the crystal energy landscape which is a projection of sampled *U*(**X**, **H**) for local minimum polymorphs and their respective densities.

Efficient, accurate and thorough sampling of  is the goal of crystal structure prediction. Historically there are many approaches to do this that go beyond what is necessary to understand this report, however the interested reader can consult the following articles (Reilly *et al.*, 2016 ▸; Nyman & Day, 2015 ▸; Schneider *et al.*, 2016 ▸; Chan *et al.*, 2021 ▸; Bier *et al.*, 2021 ▸; Yang & Day, 2021*b* ▸; Gavezzotti, 2006 ▸; Hoja *et al.*, 2017 ▸; van Eijck & Kroon, 1999 ▸; Zhu *et al.*, 2014 ▸).

In the extended variable coupled to crystal polymorph (EVCCP) scheme, a reference coordinate system with an extended variable (EV) space **S** is introduced and included as an integration variable in the partition function. **S** also represents the same CV coordinates of the molecule as **X**. In this study, the reference system is not self-interacting so *U*(**S**) = 0.

Energetic coupling between the two systems is introduced which can be written as

where

Equation (16) represents the partition function of a system that couples variables **X**, **H**, and **S**. This coupling is achieved by introducing an additional harmonic spring term with coupling strength *k* that restrains **X** and **S** to take on similar values. This coupling introduces interactions between the different variables, and β_**S**_ represents the temperature associated with the thermal bath of **S**.

However, it is crucial to emphasize that equation (16) does not imply an adiabatic separation between the systems, and the potential energy surface (PES) *U*(**X**, **H**, **S**) is distinct from *U*(**X**, **H**). Adiabatic separation would require that the evolution of **X** and **H** is completely decoupled from **S**, meaning that they evolve at different time and frequency regimes.

To handle this, the conditional probability distribution *P*(**X**, **H**|**S**) is introduced. This distribution represents the probability of observing a specific configuration of **X** and **H** given a fixed value of **S**. The existence of this conditional probability distribution indicates that **X** and **H** evolve in a way that is co-dependent with **S**, which allows for the possibility of an adiabatic separation. This conditional distribution relates to the overall joint probability distribution *P*(**X**, **H**, **S**) through the product rule of probability theory, as shown in equation (18). In general this is known as the factorization of a joint probability and equation (16) represents this in terms of a partition function which incorporates an interaction term between co-dependent variables.

Also, because

implies the relation

can be defined.

This conditional probability distribution is a subset of the larger overall distribution *P*(**X**, **H**), from which useful information and properties are extracted. In essence, the introduction of *P*(**X**, **H**|**S**) within the context of equation (16) makes the goal of sampling and extracting these useful ensemble properties more tractable (see Fig. 15 ▸). It serves to handle the interactions and dependencies between variables within the system. The notation *U*(**X**, **H**|**S**) indicates a modified potential that accounts for the adiabatic decoupling of variables for the relations described in equation (20).

This approach, where variables can now be adiabatically separated and are still co-dependent, shares conceptual similarities with umbrella sampling (Torrie & Valleau, 1977 ▸), a technique used to enhance the sampling of specific regions of the configurational space.

It is possible to state the following:

The *P*(**S**) in equation (21) is a familiar sum rule from probability theory. This means that

It is then proposed that the joint probability from equation (21) may be represented as

*i.e.**U*(**X**, **H**, **S**) is replaced with *U*(**X**, **H**|**S**) and **S** is introduced in the denominator of equation (22) as an integration variable. Note that the modified potential *U*(**X**, **H**|**S**) still remains undefined. The denominator represents another partition function  which now includes the same integration variables as equation (16).

Also the independent construction of

is made.  represents a micro-canonical partition function which will be used as a tool at a later stage. The 〈*U*_adb_(**X**, **H**|**S**)〉_*M*_ represents the average over a subset of *M* microstates, which in practice is estimated from the biased distribution *P*(**X**, **H**|**S**), *i.e.* fixed value of **S**. Equation (25) represents scanning over the entire phase space of **X** and **H** to identify all configurations where *U*(**X**, **H**) = 〈*U*_adb_(**X**, **H**|**S**)〉_*M*_. This partition function differs from a conventional micro-canonical definition in that it is representative of a ‘Dirac comb’ that identifies the subset of configurations which match for 〈*U*_adb_(**X**, **H**|**S**)〉_*M*_. The subscript adb is for ‘adiabatic’ and is used to differentiate *U*_adb_(**X**, **H**|**S**) from  =  which will be described later. One can also represent the difference between  and  as

and

The following reformulation of equation (16) into equation (24) by application of equation (25) is made. This is written out as

which is the general form of the partition function used for EVCCP. Equation (28) can be written as

where 〈**X**〉_*M*_ denotes the average value of **X** that was obtained from configurations where the value of **S** was fixed, hence **X** and **H** are adiabatically decoupled from **S** [*i.e.* using fixed **S** to sample an estimate for *P*(**X**, **H**|**S**) and obtain 〈*U*_adb_(**X**, **H**|**S**)〉_*M*_ and 〈**X**〉_*M*_]. Consider now that we can expand the energetic term in equation (29) as follows:

where *U*_adb_(**X**_*i*_, **H**_*i*_|**S**) is the energy of the *i*th polymorph generated from *P*(**X**, **H**|**S**) under the influence of the reference **S** and

In the last line of equation (30) the value *U*_min_ is introduced which is the energy of the lowest energy configuration with the harmonic penalty taken into account. *U*_min_ is obtained from sampling the *M* structures for fixed **S**. *U*_min_ enables further manipulation of equation (30) so that *P*(**X**, **H**|**S**) can be approximated with a Gaussian distribution as shown in Fig. 15 ▸ [*i.e.**P*(**X**, **H**|**S**) is a Gaussian distribution centered at **X**_min_ and **H**_min_)]. A Gaussian approximation can be made which is based on the square of the deviation of the variables **X**_*i*_ and **H**_*i*_ from those of the minimum energy polymorph configuration **X**_min_ and **H**_min_ that was identified in the mini-batch generation. Within the proposed Gaussian approximation equation (30) becomes

A Gaussian approximation implies that in equation (30) the 〈*U*_adb_(**X**_min_, **H**_min_|**S**)〉_*M*_ = *U*_min_. The summation is represented as an integral with a δ function and equation (32) is rewritten as follows:

Equations (28) and (29) are then rewritten as follows:

or

As shown in Fig. 15 ▸ the utility of this approach is to approximate the probability distribution *P*(**X**, **H**) with many smaller Gaussian distributions which are centered at **S**. Because **S** can deviate from **X** based on the value of the coupling constant (*k*) this means that the coupling constant also acts to adjust the spread of the Gaussian distribution which affects the resolution of how well the approximation of *P*(**X**, **H**) can be made since this approximation is based on sub-sampling many *P*(**X**, **H**|**S**). For very large values of *k* a reconstruction of *P*(**X**, **H**) from integration of *P*(**X**, **H**, **S**) would be as close as possible to the true *P*(**X**, **H**). However for smaller *k* the reconstruction of *P*(**X**, **H**) is smoothed.

### A2. Calculation of free energy differences

The following equations outline the scheme used for calculating the free energy difference between polymorph ensembles (*U*_min_, β_**S**_, *Z*′ = 2, SG = *P*2_1_2_1_2_1_) and (*U*_min_, β_**S**_, *Z*′ = 1, SG = *P*2_1_2_1_2_1_) sampled using EVCCPMC. The Zwanzig relation (Zwanzig, 1954 ▸) provides a prescription for calculating the free energy difference  from the ratio between  and  such that

where the subscript with angle brackets  denotes averaging taken with respect to the collective variables **S** trajectory generated using , *i.e.**Z*′ = 2.

It is appropriate to express such a formulation with respect to the energetic coupling terms [see equation (33)] used for EVCCPMC so that  and  are representative of

where the superscript (*a*) and (*b*) denote which ensemble had generated the particular variable, also *U*(*X*_1_, *X*_2_, *H*_12_|*S*_1_, *S*_2_), *U*(*X*_1_, *H*_1_|*S*_1_) and *U*(*X*_2_, *H*_2_|*S*_2_) are lattice energies which are scaled relative to *Z*′. Such a workflow requires generating a MC trajectory for *Z*′ = 2 to obtain  and  coordinates at a specified β_*S*_. This is followed by two separate *Z*′ = 1 re-calculations to obtain . So for *Z*′ = 1 the input *S* is fixed and comes directly from the *Z*′ = 2 calculation. The *S*^(*a*)^ will experience a field effect and forces created by *X*^(*b*)^ and *H*^(*b*)^ as it is coupled with the conditional distribution of polymorphs that can be generated using the fixed *S*^(*a*)^, *i.e.**P*(*X*^(*b*)^, *H*^(*b*)^|*S*^(*a*)^). The corresponding energy is evaluated as *U*(*X*^(*b*)^, *H*^(*b*)^|*S*^(*a*)^) + *k*(*X*^(*b*)^ − *S*^(*a*)^)^2^.

## Appendix B Further computational details

The algorithm for pseudo-random (PR) sampling of polymorphs and structure optimization was made available as part of the program *UPACK* (van Eijck & Kroon, 1999 ▸). The chosen force field parameters and charge assignments comply with the generalized amber force field (Wang *et al.*, 2004 ▸).

An initial polymorph description or ‘test structure’ as atomic positions and unit-cell parameters (Cartesian coordinates) or as CVs (**X** and **H**) is randomly sampled from a uniform distribution. The initial position in the polymorph landscape is then subjected to a density test so that the unit-cell parameters are sensible and within specified tolerances. Given the density test is satisfied, gradient descent moves are made based on the chosen force field and the structure is energy minimized and becomes a local minimum on the 0 K PES. If at any point within the optimization part workflow the structure energy goes above a certain threshold then the test structure is rejected.

In practice the *P*(**X**, **H**) associated with an identified polymorph at the local minimum depends on the boundaries associated with the gradients surrounding **X** and **H**. This probability is complex and not a uniform distribution. *P*(**X**, **H**) from PR sampling considers polymorphs as local minima on the PES and must have some dependence on the *U*(**X**, **H**). Generally, *P*(**X**, **H**) will take on a shape that is difficult to estimate even for very simple molecular systems and rarely evaluated in practice using statistical methods.
