# Supplementary material for: Polymorph sampling with coupling to extended variables: enhanced sampling of polymorph energy landscapes and free energy perturbation of polymorph ensembles
Source: Acta Crystallogr B Struct Sci Cryst Eng Mater. 2024 Oct 15;80(Pt 6):575–94. doi: 10.1107/S205252062400132X (PMC11789163; doi:10.1107/S205252062400132X)
Supplement: Supplementary file 1 [file b-80-00575-sup1.pdf]

Supporting Information

Sampling of probability distributions (*see subsection 3.1*) from PR and EVCCP. The set of $N, M = \{5, 10, 20, 100, 1000\}$ for $Z' = 1$ and $Z' = 2$. For this part of the study only a gentle coupling ($k = 2$) was applied during the EVCCP biased sampling. Upon comparision with PR sampling, the differnce is clearly a result of setting $\vec{X} = \vec{S}$.

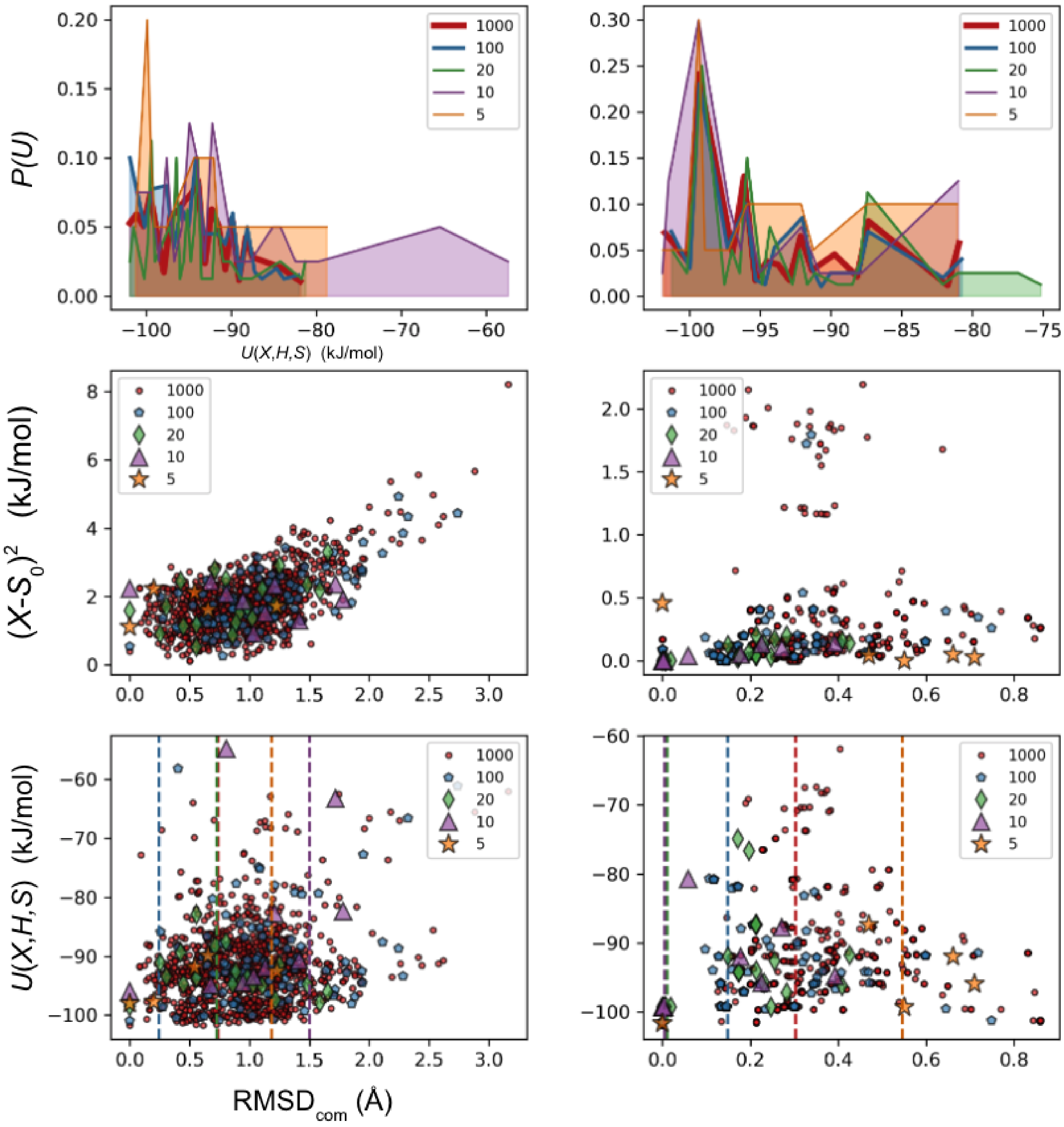

Figure S1
Plots comparing the PR(un-biased) and EVCCP(biased) coumarin $Z' = 1$ polymorph data. A different marker and colour was used to differentiate the corresponding sample size (N, M) as indicated in the legend. The $\vec{S}_0$ coordinate is that of form V. The layout is analogous to **figure 4.** in the main article.

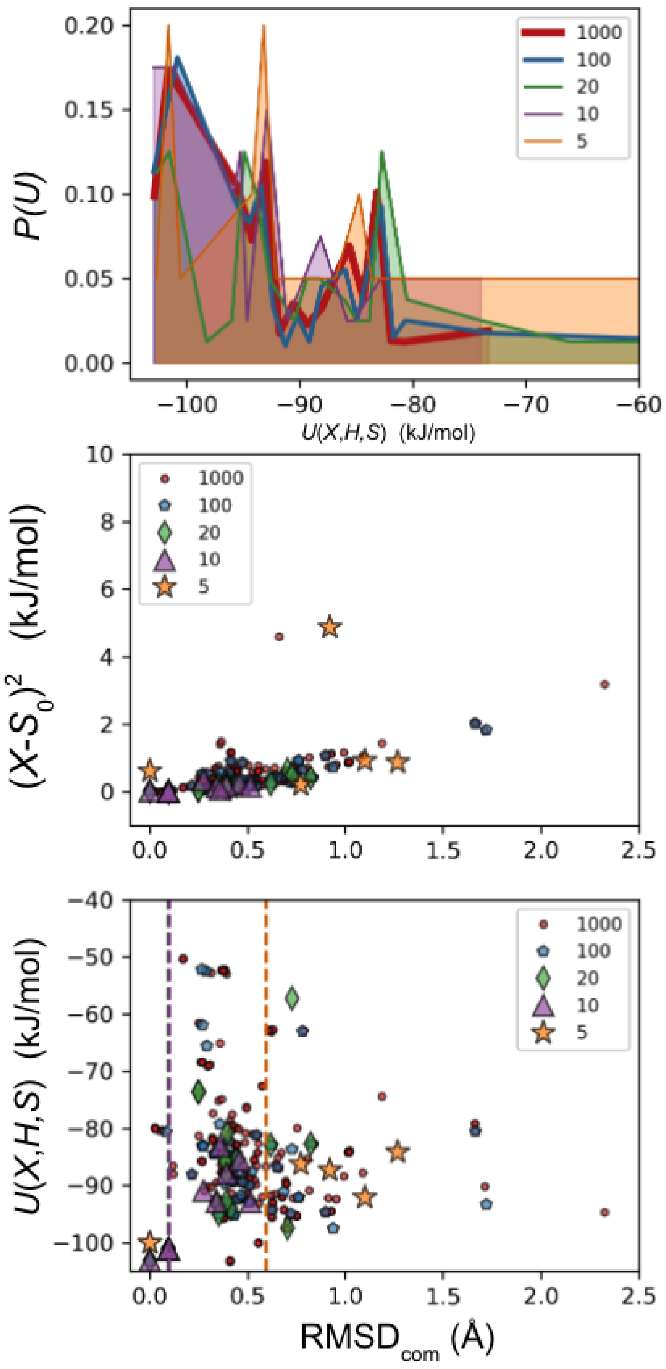

Figure S2

Plots comparing the PR(un-biased) and EVCCP(biased) coumarin $Z' = 2$ polymorph data. A different marker and colour was used to differentiate the corresponding sample size (N, M) as indicated in the legend. The $\vec{S}_0$ coordinate is that of form III. The layout is analogous to **figure 4.** in the main article.

The complete code for the experimental implementation of the python wrapper for the EVCCP methods described in the article are made available at *https://github.com/echanj/EVCCPMRE.git*. Also available are test models which run on 1D and 2D PES with general implementations. (*https://github.com/echanj/EVPT_2D_toy_models.git*)
